# Fructose-1,6-bisphosphate couples glycolytic flux to activation of Ras

**DOI:** 10.1038/s41467-017-01019-z

**Published:** 2017-10-13

**Authors:** Ken Peeters, Frederik Van Leemputte, Baptiste Fischer, Beatriz M. Bonini, Hector Quezada, Maksym Tsytlonok, Dorien Haesen, Ward Vanthienen, Nuno Bernardes, Carmen Bravo Gonzalez-Blas, Veerle Janssens, Peter Tompa, Wim Versées, Johan M. Thevelein

**Affiliations:** 1 Laboratory of Molecular Cell Biology, Institute of Botany and Microbiology, KU Leuven, Kasteelpark Arenberg 31, Leuven-Heverlee, Flanders B-3001 Belgium; 2 Center for Microbiology, VIB, Kasteelpark Arenberg 31, Leuven-Heverlee, Flanders B-3001 Belgium; 3 VIB-VUB Center for Structural Biology, Pleinlaan 2, Brussels, 1050 Belgium; 4 Structural Biology Brussels (SBB), Vrije Universiteit Brussel, Pleinlaan 2, Brussels, 1050 Belgium; 5 Laboratory of Protein Phosphorylation and Proteomics, Department of Cellular and Molecular Medicine, KU Leuven, Gasthuisberg O&N1, Herestraat 49, Leuven, 3000 Belgium

## Abstract

Yeast and cancer cells share the unusual characteristic of favoring fermentation of sugar over respiration. We now reveal an evolutionary conserved mechanism linking fermentation to activation of Ras, a major regulator of cell proliferation in yeast and mammalian cells, and prime proto-oncogene product. A yeast mutant (*tps1∆*) with overactive influx of glucose into glycolysis and hyperaccumulation of Fru1,6bisP, shows hyperactivation of Ras, which causes its glucose growth defect by triggering apoptosis. Fru1,6bisP is a potent activator of Ras in permeabilized yeast cells, likely acting through Cdc25. As in yeast, glucose triggers activation of Ras and its downstream targets MEK and ERK in mammalian cells. Biolayer interferometry measurements show that physiological concentrations of Fru1,6bisP stimulate dissociation of the pure Sos1/H-Ras complex. Thermal shift assay confirms direct binding to Sos1, the mammalian ortholog of Cdc25. Our results suggest that the Warburg effect creates a vicious cycle through Fru1,6bisP activation of Ras, by which enhanced fermentation stimulates oncogenic potency.

## Abstract (web summary)

Yeast and cancer cells both favor sugar fermentation in aerobic conditions. Here the authors describe a conserved mechanism from yeast to mammals where the glycolysis intermediate fructose-1,6-bisphosphate binds Cdc25/Sos1 and couples increased glycolytic flux to increased Ras proto-oncoprotein activity.

## Introduction

Yeast is one of the most prominent examples of eukaryotic cells, which displays under aerobic conditions high fermentative activity as well as rapid cell proliferation, just like mammalian cancer cells^1^. Warburg suggested that high glycolytic activity may be causally related to the cancerous state^2^, while in yeast high glycolytic activity also correlates with the most rapid cell proliferation. There is a striking correlation between the rate of uncontrolled cell proliferation, the aggressive metastasis character of cancers and the extent of the ‘Warburg effect’^3, 4^. In spite of many studies, however, it remains controversial whether strong fermentation is a cause or a symptom of cancer since no clear molecular link between glycolysis and proteins controlling cell proliferation has been identified^5^.

**Fig. 1:**
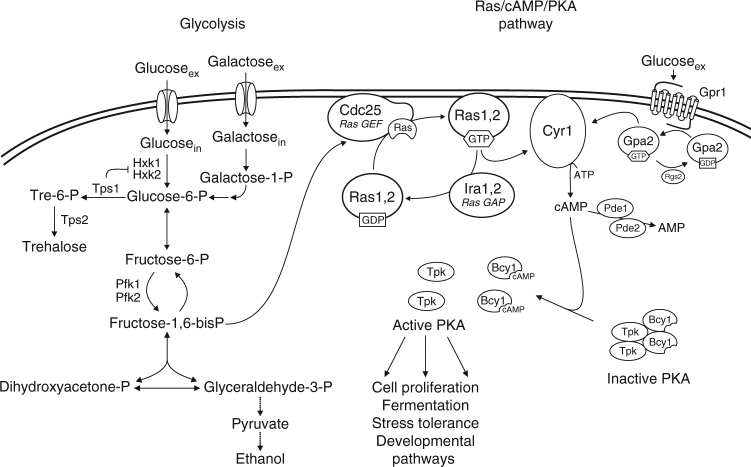
Schematic overview of initial glucose metabolism in yeast and its connection to activation of the Ras-cAMP-PKA pathway. Extracellular glucose is transported by facilitated diffusion into the cells after which it is phosphorylated by one of the two hexokinases or by glucokinase. The activity of the two hexokinases, but not glucokinase, is feedback-inhibited by Tre6P, the intermediate in trehalose biosynthesis. Glu6P is further converted into Fru6P, Fru1,6bisP, the triose phosphates DHAP and GAP, and the latter is subsequently converted to pyruvate and the fermentation product ethanol. Galactose is taken up by its own carrier, phosphorylated to Gal1P and further converted into Glu6P, where it joins the glucose catabolic pathway, effectively bypassing the hexokinase catalyzed step in glycolysis. The Ras-cAMP-PKA pathway exerts major control over cell proliferation, fermentation rate, stress tolerance and developmental pathways in yeast. As in mammalian cells, cAMP is synthesized by adenylate cyclase (Cyr1) and binds to the Bcy1 regulatory subunits of PKA, causing their dissociation from the catalytic Tpk subunits with activation of the latter as result. Yeast adenylate cyclase is activated by a glucose-sensing G-protein coupled receptor (GPCR) system, composed of the Gpr1 receptor, the Gα protein Gpa2, and its GAP factor Rgs2, similar to activation of adenylate cyclase by GPCR systems in mammalian cells. As opposed to mammalian cells, however, yeast adenylate cyclase is also activated by the Ras1,2 proteins, which are controlled by the Cdc25 (and Sdc25) guanine nucleotide exchange factor (GEF) and the GTPase activating proteins (GAP) Ira1,2. Ras and its regulators are highly conserved between yeast and mammalian cells (the GEF Sos and the GAP NF1, respectively). Although it was known that glucose catabolism in glycolysis is required for glucose activation of the Ras proteins and cAMP synthesis, the molecular connection remained unknown. In the present paper, it is shown that Fru1,6bisP functions as activator of Ras by interacting with Cdc25 and that this connection between glycolysis and Ras is conserved between yeast and mammalian cells

Yeast and mammalian cells also share the G-protein and oncogene product, Ras, with a homologous set of regulators, Cdc25,Sdc25/Sos1 and Ira1,2/NF1, but with different target proteins, adenylate cyclase in yeast and, e.g., the Raf protein kinase in mammalian cells^6–8^. Ras is highly conserved. Mammalian Ras can functionally replace yeast Ras^9^ and hyperactive Ras oncogenes cause aberrant cell proliferation control and apoptosis induction in yeast^10, 11^. Mutant *RAS* genes, encoding overactive (permanently GTP loaded) Ras proteins, are among the most common oncogenes found in naturally occurring and artificially induced cancer cells^12^. Ras also serves as an important regulator of cell proliferation in yeast acting through activation of cAMP synthesis and the protein kinase A (PKA) pathway (Fig. 1)^6^. Glucose is a potent activator of cAMP synthesis and thus of protein kinase A (PKA) in yeast, and this is mediated by Cdc25/Ras in concert with a glucose-sensing G-protein coupled receptor system (Fig. 1)^13–18^. Glucose catabolism in glycolysis is required for cAMP signaling and activation of the Ras proteins (Fig. 1)^15, 19^ but the underlying mechanism has been unclear. The strong conservation of Ras and its regulators, Cdc25,Sdc25/Sos1 and Ira1,2/NF1, in yeast and mammalian cells, suggests that the unknown mechanism responsible for glycolytic activation of Ras may also be conserved in the two cell types.

In nearly all cells, glucose is phosphorylated by hexokinase or glucokinase to glucose-6-phosphate (Glu6P) after its transport into the cells, then converted to fructose-6-phosphate (Fru6P) by phosphoglucoisomerase and subsequently phosphorylated to Fru1,6bisP by phosphofructokinase 1 (Fig. 1). Fru1,6bisP is by far the most elaborately controlled glycolytic metabolite since its biosynthetic and hydrolytic enzymes are post-translationally controlled by multiple mechanisms, including a specific allosteric regulator, Fru2,6bisP, which is synthesized and hydrolyzed in a parallel regulatory pathway^20^. Fru1,6bisP is split into the triose phosphates dihydroxyacetone phosphate (DHAP) and glyceraldehyde-3-phosphate (GAP), which are ultimately converted into pyruvate, and further into a fermentation product, either ethanol in yeast (Fig. 1) or lactic acid in mammalian cells.

Multiple molecular changes have been identified in cancer cells contributing to the high glycolytic rate, including enhanced intrinsic activity of phosphofructokinase 1 and higher levels of its allosteric activator Fru2,6bisP^3, 21, 22^. Hexokinase activity has also received particular attention as one of the major determinants of the Warburg effect. In certain types of cancer cells, type II hexokinase is strongly associated with the mitochondrial ADP/ATP carrier, losing its feedback inhibition by glucose-6P and allowing much higher catalytic activity due to efficient coupling with mitochondrial ATP provision^3^. When such cancer cells were grown on galactose, a sugar whose metabolism does not involve hexokinase, the high fermentation rate was strongly reduced^23^. Subsequent work showed that this alteration of type II hexokinase was sufficient to enhance the glycolytic rate of a normal cell up to that observed in cancer cells^24^. Extensive additional evidence has highlighted the critical role of type II hexokinase in aberrant glycolytic metabolism of cancer cells^3^.

Whereas mammalian hexokinase is feedback-inhibited by its product Glu6P^25^, yeast hexokinase is feedback inhibited by trehalose-6-phosphate (Tre6P) (Fig. 1)^26^. Tre6P is synthesized from Glu6P and UDPG by Tre6P synthase (Tps1) as intermediate of trehalose biosynthesis (Fig. 1). *Tps1∆* cells have unbridled hexokinase activity and display uncontrolled influx of glucose into glycolysis^27^. This is characterized by hyperaccumulation of metabolites upstream of glyceraldehyde-3-phosphate dehydrogenase (GAPDH) in the upper part of glycolysis, especially Fru1,6bisP, and rapid depletion of ATP, phosphate and glycolytic metabolites downstream of GAPDH. As a result the *tps1∆* strain is unusually sensitive to low amounts (a few mM) of glucose^28, 29^. Deletion of hexokinase 2, the most active hexokinase isoenzyme in yeast, completely suppresses aberrant glucose catabolism and restores growth on glucose of the *tps1∆* strain^30^. Since galactose metabolism uses galactokinase rather than hexokinase for conversion of galactose into Glu6P and entry at this point into glycolysis, *tps1∆* cells do not show deregulated glycolysis with galactose as substrate and grow normally on galactose. Hence, in a certain sense, *tps1∆* cells resemble cancer cells with unrestricted hexokinase activity, although their fermentation defect is much more severe, causing complete absence of growth on glucose.

In the present work, we have made use of the yeast *tps1∆* mutant to identify the molecular connection between glucose fermentation and activation of Ras. We provide extensive evidence for an evolutionary conserved mechanism between yeast and mammalian cells in which Fru1,6bisP functions in physiological concentrations as a potent activator of the Ras proteins, acting through its Cdc25/Sos1 guanine nucleotide exchange factor. This finding suggests that the Warburg effect, i.e., the elevated fermentation rate in cancer cells, creates a vicious cycle through Fru1,6bisP activation of Ras, causing enhanced fermentation rate to stimulate oncogenic potency.

## Results

### Glucose triggers apoptosis through Ras

**Fig. 2:**
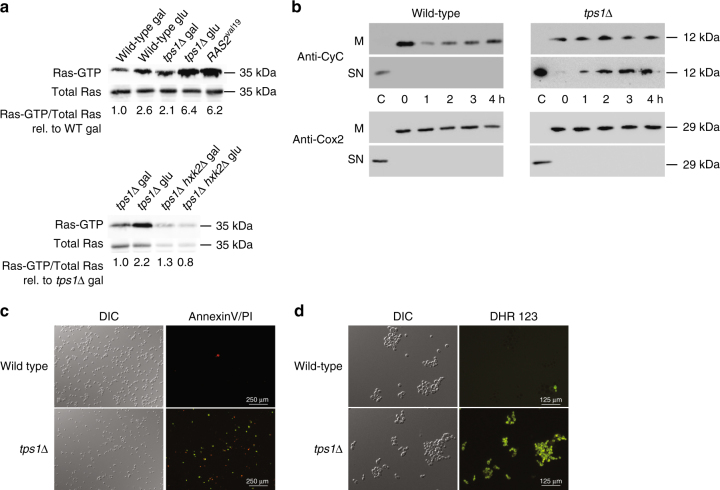
Glucose triggers activation of Ras and apoptosis in *tps1∆* cells. **a** Ras-GTP level before and 10 min after addition of glucose (glu) to cells grown on galactose (gal). The strain expressing *Ras2* ^*val19*^ was grown on glucose. Total Ras = Ras-GDP + Ras-GTP. Quantification of the signals as Ras-GTP level/total Ras level compared to the ratio for wild type or *tps1∆* on galactose set at 1.0. The values were determined through quantification by densitometry and are expressed as relative arbitrary units. **b** Specific cytochrome c release from the mitochondria after addition of glucose to wild type and *tps1∆* strain. M, mitochondrial fraction; SN, post-mitochondrial supernatant; Anti-CyC, anti-cytochrome *c* antibody; Anti-Cox2, cytochrome oxidase 2 antibody; C: control cytochrome c or cytochrome oxidase 2. **c** Annexin-V-FITC/propidium iodide staining 2h after addition of glucose to cells grown on galactose. Green colored fluorescence originates from annexin staining, red colored fluorescence represents propidium iodide binding to DNA of death cells. The scale bar is 250 μm. **d** Reactive oxygen species (ROS) accumulation 2 h after addition of glucose to cells grown on galactose. ROS was stained with the dye dihydrorhodamine 123. DIC, differential interference contrast image. The scale bar is 125 μm

We show that glucose addition to galactose-grown *tps1∆* cells triggers rapid and dramatic activation of Ras, as opposed to the modest activation observed in wild type cells (Fig. 2a). The GTP loading state on Ras in the *tps1∆* strain, 10 min after glucose addition, is similar to that in a strain expressing the constitutively active Ras2^val19^ oncogene equivalent (Fig. 2a). A Ras2^val19^ strain, however, has no detectable growth inhibition on glucose medium^10^. Deletion of hexokinase 2 abolishes glucose-induced hyperactivation of Ras (Fig. 2a), which is consistent with its complete suppression of the glucose growth defect of the *tps1∆* strain^30^. Whereas the Ras2^val19^ strain shows normal growth on glucose, it grows with strongly reduced rate on poor carbon sources, like galactose, and dies in stationary phase^10^. More recent work has shown that hyperactive Ras causes programmed cell death/apoptosis in non-growing yeast cells^11^. Hence, we reasoned that the combination of aberrant growth initiation due to the glycolytic deregulation and hyperactivation of Ras in *tps1∆* cells may cause apoptosis and that this actually might be the real cause for the unusually high glucose sensitivity of *tps1∆* cells^29^ rather than the deregulation of glycolysis per se. Determination of classical read-outs for apoptosis in yeast gave results consistent with this hypothesis. Glucose addition to *tps1∆* cells, as opposed to wild type cells, caused rapid release of cytochrome c from the mitochondria (Fig. 2b), exposure of phosphatidylserine at the plasma membrane (Fig. 2c) and generation of reactive oxygen species (ROS) (Fig. 2d). These results are consistent with recent evidence that the Tps1 protein acts as an anti-apoptotic factor in yeast^31^. The drop in the cytochrome c level in the wild-type cells might be caused by glucose repression of the *CYC1* gene^32^. It is absent in the *tps1∆* strain because that strain is deficient in glucose-induced signaling^28^. The inability to detect a decrease in the cytochrome *c* content of the mitochondria in the *tps1∆* strain concomitant with the appearance of cytochrome c in the supernatant, is likely due to the fact that the amount appearing in the supernatant is much smaller than the amount remaining in the mitochondria.

**Fig. 3:**
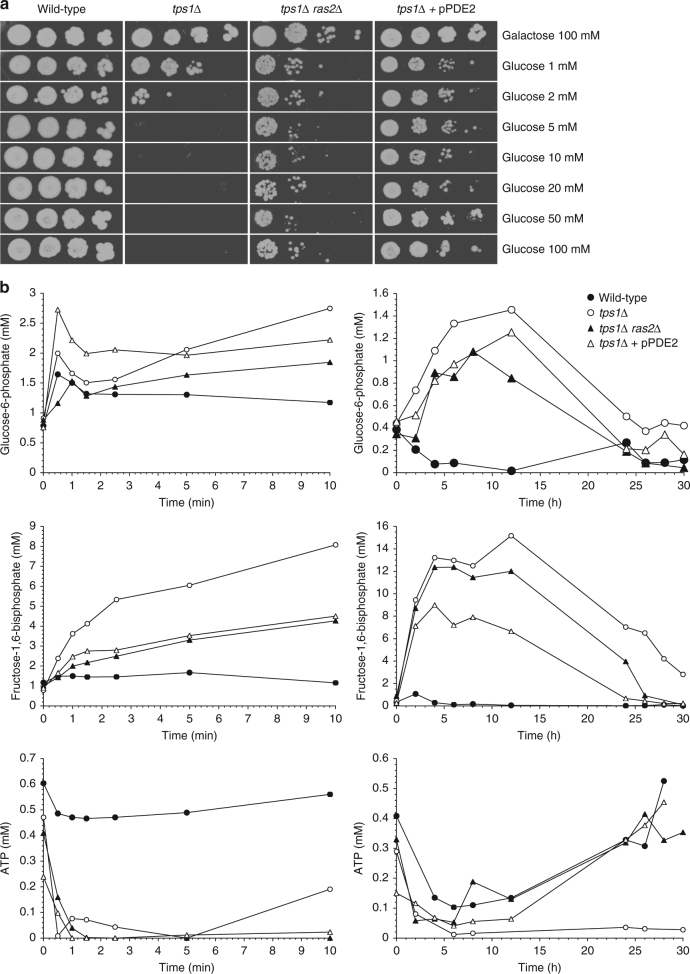
Restoration of growth on glucose and glycolytic metabolite levels in the *tps1∆* strain with downregulation of the Ras-cAMP-PKA pathway. **a** Growth of *tps1∆* and suppressor strains on galactose and different concentrations of glucose. Five-fold dilutions were spotted. **b** Intracellular Glu6P, Fru1,6bisP and ATP concentration before and after addition of 100 mM glucose. The short-term measurements (0–10 min) were performed with non-growing cells incubated in 25 mM MES buffer (pH 6.0), while the long-term measurements (0–30 h) were performed with cells growing in minimal galactose medium

Deletion of Ras2 in the *tps1∆* strain caused partial recovery of growth on glucose (Fig. 3a). Overexpression of *PDE2*, which encodes the high-affinity cAMP phosphodiesterase and is known to counteract phenotypes caused by hyperactive Ras^33^, restored growth on glucose even better (on solid medium: Fig. 3a; in liquid medium: Supplementary Fig. 1). This may be due to the fact that overexpression of Pde2 lowers the cAMP level in the cells even more than deletion of Ras2 and thus leads to lower PKA activity. Our results are consistent with overactive Ras playing a role in the glucose growth defect of the *tps1∆* strain. Interestingly, both in the *tps1∆* and *tps1∆ ras2∆* strain a very similar deregulation of glycolysis after addition of glucose was present, including hyperaccumulation of Fru1,6bisP and depletion of ATP, but the metabolite profile started to recover in the *tps1∆ ras2∆* strain after about 5 h to finally reach the same profile as observed in wild type cells (Fig. 3b). The difference was most striking in the ATP level, which stayed close to zero in the *tps1∆* strain, but recovered to the same level as in wild type cells in the *tps1∆ ras2∆* and *tps1∆* p*PDE2* strains (Fig. 3b). This indicates remarkable robustness and flexibility of the glycolytic pathway in yeast and contradicts that the strong perturbation of metabolite homeostasis is causing the glucose growth defect. The results suggest that the induction of programmed cell death, caused by overactivation of the Ras-cAMP-PKA pathway, is the true cause of the inability of the *tps1∆* strain to grow on glucose, rather than the glycolytic deregulation, which is usually thought to be the main cause^34^. The onset of the normalization of glycolytic metabolite homeostasis a few hours after addition of glucose in the *tps1∆ ras2∆* and *tps1∆* p*PDE2* strains (Fig. 3b) might suggest that an additional time-dependent change in initial glucose catabolism occurs, which allows the cells to recover normal glycolytic flux and intermediate levels.

### Fru1,6bisP triggers activation of Ras

**Fig. 4:**
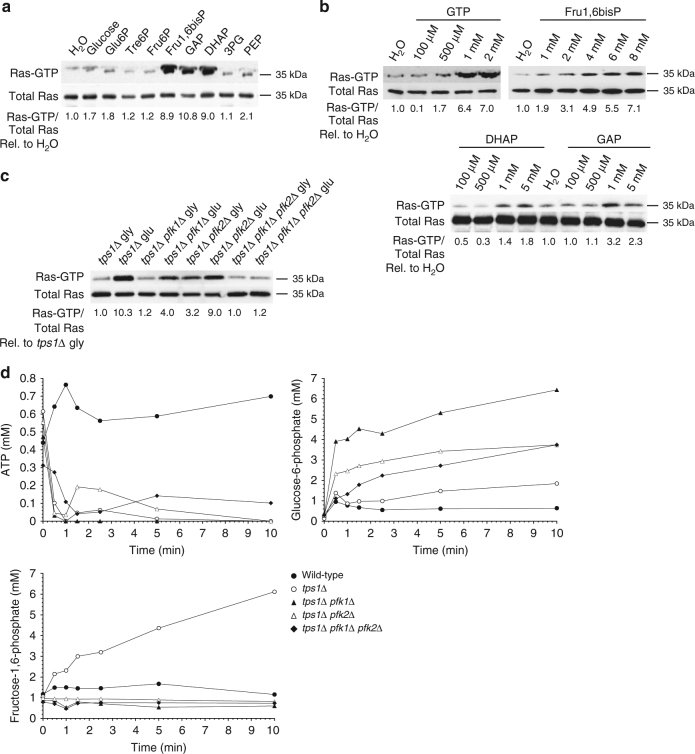
Activation of Ras by Fru1,6bisP. **a** Ras-GTP level 3 min after addition of 5 mM of different glycolytic metabolites to permeabilized yeast spheroplasts. Glu6P, glucose-6-phosphate; Tre6P, trehalose-6-phosphate; Fru6P, fructose-6-phosphate; Fru1,6bisP, fructose-1,6-bisphosphate; DHAP, dihydroxyacetone phosphate; GAP, glyceraldehyde-3-phosphate; 3PG, 3-phosphoglycerate; PEP, phosphoenolpyruvate. Quantification of the signals as Ras-GTP level/total Ras level compared to the ratio for the water control set at 1.0. The values were determined through quantification by densitometry and are expressed as relative arbitrary units. **b** Activation of Ras in permeabilized spheroplasts with different concentrations of GTP, Fru1,6bisP, DHAP and GAP. Quantification of the signals as Ras-GTP level/total Ras level compared to the ratio for the water control set at 1.0. The values were determined through quantification by densitometry and are expressed as relative arbitrary units. **c** Ras-GTP level before and 10 min after addition of 100 mM glucose (glu) to cells grown on glycerol +2.5 mM glucose (gly). Quantification of the signals as Ras-GTP level/total Ras level compared to the ratio for *tps1∆* on glycerol set at 1.0. The values were determined through quantification by densitometry and are expressed as relative arbitrary units. **d** Intracellular ATP, Glu6P, and Fru1,6bisP concentration before and after addition of 100 mM glucose to cells grown in glycerol +2.5 mM glucose

To investigate whether one or more of the hyperaccumulated glycolytic metabolites in the *tps1∆* strain acted as the trigger for hyperactivation of Ras, we made use of nystatin-permeabilized wild type yeast spheroplasts^35^. These are cells of which the cell wall has been largely removed and the plasma membranes permeabilized to allow passage of all small molecules. Because of their fragility the permeabilized cell sacs were not washed and the small molecules leaked into the medium were thus only strongly diluted. Addition of glycolytic metabolites to permeabilized spheroplasts revealed strong activation of Ras by Fru1,6bisP at the higher physiological concentrations (4–8 mM; Fig. 4a, b), similar to that observed with physiological concentrations of GTP (Fig. 4b). DHAP and GAP also caused activation but only in unphysiologically high concentrations (1–5 mM; Fig. 4a, b). Other glycolytic intermediates, and glucose or Tre6P did not cause activation (Fig. 4a).

To evaluate whether Fru1,6bisP is responsible for glucose-induced hyperactivation of Ras in the *tps1∆* strain, we constructed *tps1∆* strains with additional single or double deletion of the *PFK1* and *PFK2* genes, encoding phosphofructokinase 1. Glucose-induced activation of Ras was reduced in cells of the *tps1∆ pfk1∆* and *tps1∆ pfk2∆* strains, and abolished in cells of the *tps1∆ pfk1∆ pfk2∆* strain (Fig. 4c). This would be consistent with the hyperaccumulation of Fru1,6bisP in *tps1∆* cells being responsible for hyperactivation of Ras. However, upon measurement of the glycolytic metabolites, the *tps1∆ pfk1∆* and *tps1∆ pfk2∆* strains were already deficient in glucose-induced hyperaccumulation of Fru1,6bisP and consistently showed a much higher accumulation of Glu6P than the *tps1∆* strain (Fig. 4d). Glu6P was also higher than in the corresponding *pfk1∆* and *pfk2∆* strains (Fig. 4d).

The presence of partial Ras activation without Fru1,6bisP accumulation in the *tps1∆ pfk1∆* and *tps1∆ pfk2∆* strains suggests that there may be a second mechanism responsible for glucose-induced hyperactivation of Ras in the *tps1∆* strain and/or that the hyperaccumulation of Glu6P in these strains in some way triggers a residual activation. Although the complete absence of Ras hyperactivation in the *tps1∆ pfk1∆ pfk2∆* strain seems to contradict this possibility, the very poor growth of this strain, requiring feeding with both a respiratory carbon source and a low level of glucose, may have compromised the second activation mechanism. It can also not be excluded that one of the glucose signaling pathways modifies Cdc25, for instance through phosphorylation, to render it more sensitive to Fru1,6bisP. This could explain why even in the absence of an increase in the level of Fru1,6bisP, it would still be able to trigger activation of Ras after addition of glucose.

### Fru1,6bisP acts through the Ras GEF Cdc25 in yeast

**Fig. 5:**
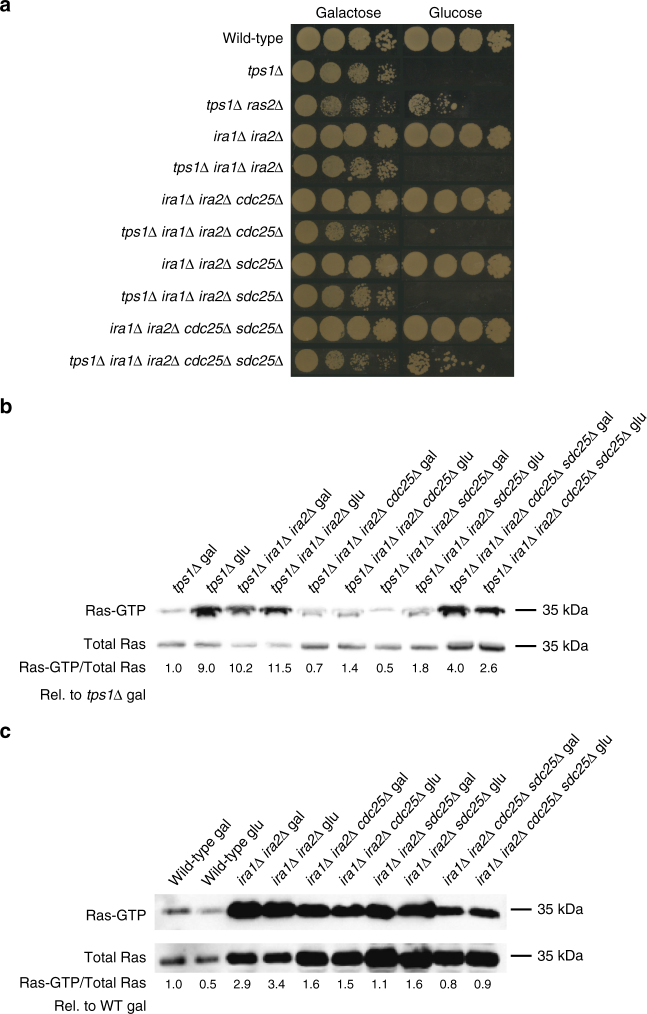
Evaluation of requirement for the GEF and GAP regulators of Ras. **a** Growth on galactose and glucose. Five-fold dilutions were spotted. **b**, **c** Ras-GTP level before and 10 min after addition of glucose (glu) to cells grown on galactose (gal). Quantification of the signals as Ras-GTP level/total Ras level compared to the ratio for wild type (**b**) or *tps1∆* (**c**) on galactose set at 1.0. The values were determined through quantification by densitometry and are expressed as relative arbitrary units

We next investigated whether Fru1,6bisP caused direct activation of Ras or acted through one of its regulators, the guanine nucleotide exchange proteins Cdc25 and Sdc25, or the GTPase activating proteins Ira1 and Ira2. For that purpose we constructed several strains with combinations of *tps1∆* and deletions in the genes encoding these regulators. Deletion of both *CDC25* and *SDC25* is lethal^36^, while deletion of *IRA1* and *IRA2* causes hyperactivation of Ras^37^. The *ira1∆ ira2∆ cdc25∆ sdc25∆* strain retains enough Ras activity for growth (Fig. 5a). All quadruple *tps1∆* strains with at least one wild-type GEF factor, Cdc25, or Sdc25, were unable to grow on glucose, whereas absence of both GEF factors in the quintuple deletion strain *tps1∆ cdc25∆ sdc25∆ ira1∆ ira2∆* restored to some extent growth on glucose medium (Fig. 5a, Supplementary Fig. 2). This suggests that glucose-induced hyperactivation of Ras through the GEF factors is a major cause of the glucose sensitivity of the *tps1∆* strain. The *tps1∆ ira1∆ ira2∆* strain showed already a high Ras-GTP level on galactose medium, which made it difficult to evaluate any further glucose-induced increase (Fig. 5b). On the other hand, in the quadruple deletion strains, *tps1∆ sdc25∆ ira1∆ ira2∆* and *tps1∆ cdc25∆ ira1∆ ira2∆*, the rapid glucose-induced hyperactivation of Ras was largely abolished, indicating requirement of both GEF factors. The absence of Ras hyperactivation in these strains may be restricted to the short-term response, since both strains were still unable to grow on glucose (Fig. 5a). Unexpectedly, we found that the quintuple deletion strain, *tps1∆ cdc25∆ sdc25∆ ira1∆ ira2∆*, showed a higher basal activity of Ras on galactose medium, which, however, did not increase further upon addition of glucose (Fig. 5b). Although these experiments and the interpretation of the results are complicated because of the essential character of the GEF factors, the high basal Ras activity caused by deletion of the GAP proteins, and the unexpectedly higher basal level of Ras-GTP in the quintuple deletion strain, the results are consistent with a role of the GEF factors in mediating glucose-induced hyperactivation of Ras in the *tps1∆* strain. This was confirmed by the observation that deletion of *SDC25* and especially *CDC25* in the *tps1∆* strain caused partial growth recovery on glucose (Supplementary Fig. 3). This result also suggests that Fru1,6bisP exerts its activating effect mainly through Cdc25.

We have also performed similar experiments in the wild-type strain background. This confirmed the much weaker and more variable glucose-induced activation of Ras in the wild type strain (which may be due to transient oscillation in the response; Fig. 5c). Deletion of *IRA1* and *IRA2* caused a dramatic increase in Ras-GTP both in galactose and glucose incubated cells (Fig. 5c), which made it impossible to evaluate reliably any possible further increase in Ras-GTP in the presence of glucose. Deletion of *CDC25* or *SDC25* caused a partial reduction of the very high Ras-GTP level, while double deletion of *CDC25* and *SDC25* caused a further reduction in the Ras-GTP level (Fig. 5c). In none of the latter strains was a significant difference between the galactose and glucose condition observed, consistent with the idea that the glucose activation effect on Ras acts through its Cdc25 and Sdc25 GEF factors.

**Fig. 6:**
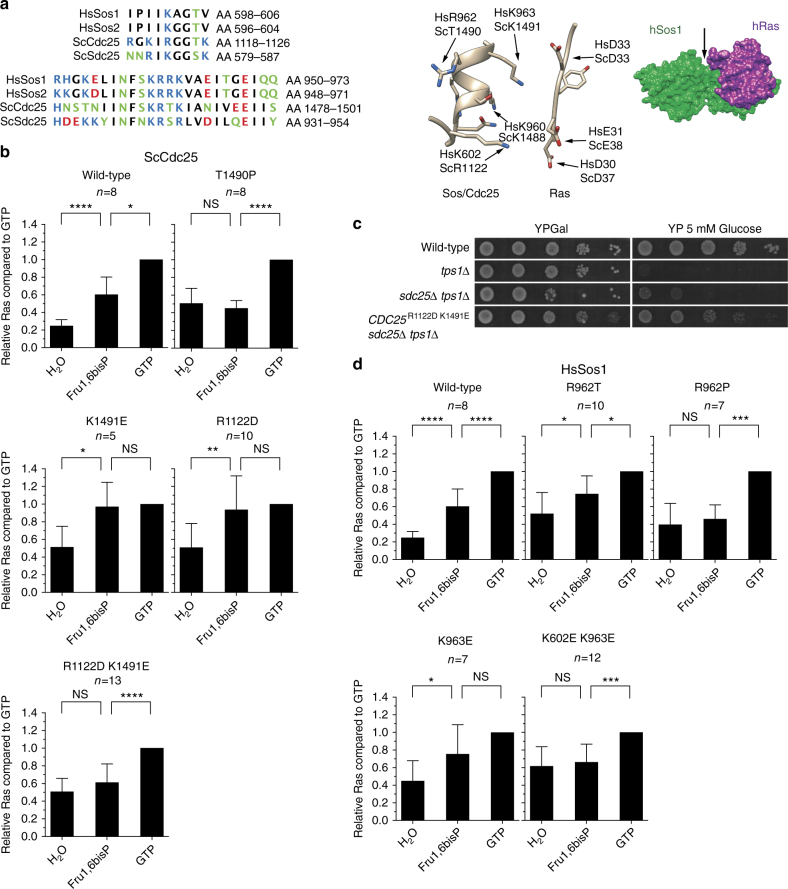
Conserved residues in the C terminus of Cdc25 and Sos1 are required for Fru1,6bisP activation of Ras. **a** Alignment of the conserved region in the Ras GEF factors, HsSos1 and HsSos2 from *Homo sapiens* and ScCdc25 and ScSdc25 from *Saccharomyces cerevisiae*. Crystal structure of the complex between human Sos1 (green) and human Ras (purple) (PD entry: 1BKD^40^). The arrow points to the cleft, which contains the conserved region. Enlarged view on the 3D structure surrounding the relevant residues in Sos1 and Cdc25 located in the conserved region. **b**, **d** Ras-GTP level 3 min after addition of 5 mM Fru1,6bisP or 1 mM GTP as positive control, to permeabilized yeast spheroplasts from strains expressing mutant ScCdc25 (**b**) or HsSos1 (**d**) alleles. Western blots were quantified and the Ras-GTP levels are shown relative to the level observed after addition of GTP. Values represent average (number of replicates shown above each graph), error bars represent standard deviations. Sidak’s multiple comparison test was used for evaluation of statistical significance ((NS) identifies *p*-values greater than 0.05, (*) identifies *p*-values between 0.01 and 0.05, (**) between 0.01 and 0.001, (***) between 0.001 and 0.0001, (****) identifies p-values lower than 0.0001). **c** Recovery of growth on glucose in the *tps1∆ sdc25∆* strain expressing Cdc25^R1122D,K1491E^ instead of wild type Cdc25

Since previous work had shown that the highly conserved C terminus of Cdc25 was required for glucose activation of cAMP synthesis^38, 39^, we focused on this part of Cdc25 to identify amino acid residues of which mutagenesis might abolish Fru1,6bisP activation of Ras in permeabilized spheroplasts and also restore growth of the *tps1∆* mutant on glucose. We selected for site-directed mutagenesis a highly conserved region with several positively charged residues (Fig. 6a) (aa 1478–1501 in Cdc25) that could possibly be involved in the formation of a binding site for the negatively charged Fru1,6bisP molecule. Interestingly, another positively charged residue, R1122 in yeast Cdc25, which corresponds to K602 in human Sos1, resides in the vicinity of this region in the 3D structure of the human Sos1 catalytic domain^40^. The R1122/K602 residue is located itself in a small conserved region. Figure 6a shows the 3D structure of Sos1 in association with Ras and the position in space of selected amino acid residues in the cleft formed by the alpha helix I of Sos1 and the switch 1 region of Ras, thus in the area where the two proteins interact. The amino acid residues K602, R962, and K963 of Sos1, are located within this cleft (Fig. 6a).

We first mutagenized the positively charged residues R1122 and K1491 in Cdc25 to negatively charged aspartate and glutamate residues, respectively. In addition, we constructed the T1490P allele of Cdc25 in order to disturb the structure of the alpha helix^39^ in this region. In permeabilized spheroplasts of strains expressing Cdc25^R1122D,K1491E^ or Cdc25^T1490P^ instead of wild-type Cdc25, addition of Fru1,6bisP in a physiological concentration of 5 mM no longer caused activation of Ras as opposed to addition of GTP (Fig. 6b). This indicates that the residues R1122 and K1491 are in some way important for Fru1,6bisP activation of Ras, either because of direct interaction with Fru1,6bisP or maintenance of a proper Cdc25 conformation, and that also the structure of the alpha helix surrounding T1490 is important for a similar reason.

The activity of Sdc25 was assumed negligible since it is repressed during exponential growth in rich media^36^. The strains carrying the Cdc25 mutated alleles in the absence of Sdc25 were viable, showing that the alleles were functional. Interestingly, a *tps1∆ sdc25∆* strain expressing Cdc25^R1122D,K1491E^ instead of wild-type Cdc25 displayed strong recovery of growth on glucose, supporting the requirement of these residues for Fru1,6bisP activation of Ras in vivo (Fig. 6c). We did not observe such recovery of growth on glucose with a strain expressing Cdc25^T1490P^ instead of wild-type Cdc25 (Supplementary Fig. 4a). However, such a strain displayed reduced trehalose and glycogen levels, suggesting the presence of constitutively activated PKA (Supplementary Fig. 4b). This may be explained by Thr^1490^ serving as a PKA target site for feedback inhibition of PKA on Cdc25, as previously suggested^39^ or may be due to another effect caused by the distortion of the alfa-helix structure.

Next, we assessed whether human Sos1 expressed in yeast is also responsive to the same type of glycolytic stimulation as yeast Cdc25. Interestingly, addition of Fru1,6bisP in a physiological concentration of 5 mM to permeabilized yeast spheroplasts expressing only the guanine nucleotide exchange factor region of human Sos1 (residues 553–1024), caused a clear activation of Ras similar to that of GTP (Fig. 6d). Wild type Sos1 has the INFSKRR962K sequence. However, the yeast strain expressing the complete Sos1 protein grew poorly on galactose. The mutant protein R962T, which has the same INFSKRTK sequence as in Cdc25, also supported activation of Ras with Fru1,6bisP and GTP (Fig. 6d). The strain expressing this protein showed normal growth on galactose, consistent with the threonine being a target of PKA feedback inhibition. Activation of Ras in permeabilized spheroplasts with Fru1,6bisP, but not GTP, was absent for the mutant alleles Sos1^K602E, K963E^ and Sos1^R962P^ (Fig. 6d), which have the equivalent mutations to those mentioned above for yeast Cdc25^40^. The single-mutation K963E did not yet abolish Fru1,6bisP activation of Ras. These results show that Sos1 is able to mediate activation of Ras by Fru1,6bisP and that the corresponding amino acid residues or combinations to those in Cdc25 are also important for the activation.

There are several possibilities for the mechanism by which interaction of Fru1,6bisP with Cdc25/Sos1 might stimulate activation of Ras. It might alleviate one of the limiting steps in the Cdc25/Sos1-Ras nucleotide exchange cycle, for instance the binding of Ras to Cdc25/Sos1, the release of GDP, the incorporation of GTP, the dissociation of Cdc25/Sos1 from Ras or the shielding of Ras from the action of the GTPase activating proteins Ira1,2/NF1.

### Fru1,6bisP stimulates dissociation of the Ras/Sos1 complex

**Fig. 7:**
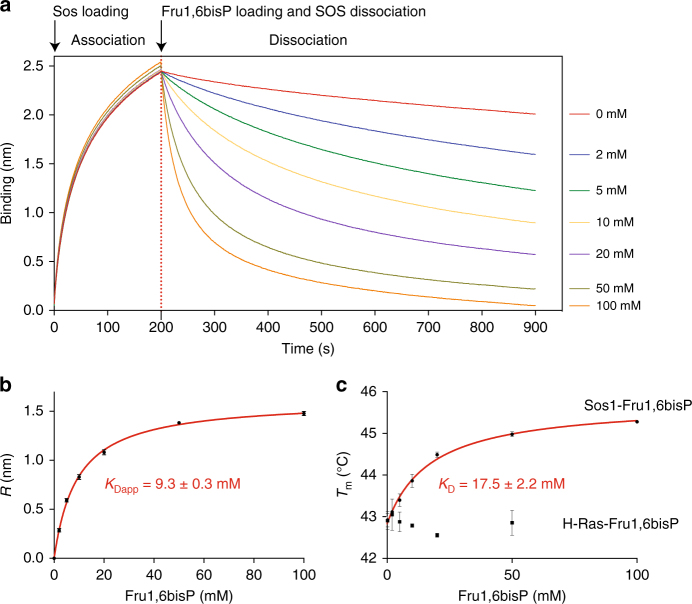
Fru1,6bisP binds to Sos1 and disrupts the Sos1/H-Ras complex. **a**, Biolayer interferometry (BLI) measurements showing the disruption of the Sos1/H-Ras complex. His-tagged H-Ras was coupled to Ni^2+^-coated biosensors and loaded with 0.5 µM of non-tagged Sos1 (association phase). Subsequently, dissociation of Sos1 was monitored in buffer containing increasing concentrations of Fru1,6bisP (0, 2, 5, 10, 20, 50, and 100 mM as labeled on the curves). The rates of Sos1 dissociation are dependent on the Fru1,6bisP concentration. **b** Titration curve showing the influence of Fru1,6bisP concentration on Sos1/H-Ras complex dissociation. Plotting the BLI signal amplitude as shown in panel **a** after 150 s of complex dissociation (*R*; mean ± s.d.; *n* = 3 independent experiments) vs. the Fru1,6bisP concentration yields a binding curve that was fitted on a Langmuir binding model to obtain an apparent *K*_D_ (*K*_Dapp_) value (±s.e.). **c** Titration curves for binding of Fru1,6bisP to either Sos1 or H-Ras. Melting temperatures (*T*_m_) of Sos1 and H-Ras at different ligand concentrations were obtained via thermal shift assays (*n* = 3 independent experiments). Plotting *T*_m_ values (mean ± s.d.) vs. ligand concentration yielded binding curves that were fitted on a Langmuir binding model to obtain *K*_D_ values (±s.e.)

To study the interaction of Fru1,6bisP with Ras and its Ras GEF factor, we performed an in vitro binding assay (Octet Biolayer interferometry) using human H-Ras (residues 1–166) and the catalytic part of Sos1 (residues 564–1049), and analyzed the effect of Fru1,6bisP on the stability of the Ras/Sos1 complex. First, H-Ras molecules were loaded onto the biosensor, and Sos1 molecules were allowed to bind to H-Ras during the association phase. Next, the dissociation of the H-Ras/Sos1 complex was monitored in the presence of increasing concentrations of Fru1,6bisP. The biolayer interferometry measurements showed that Fru1,6bisP increases the dissociation rate of the H-Ras/Sos1 complex in a dose-dependent way (Fig. 7a). Plotting of the response (taken 150 s after addition of Fru1,6bisP) vs. the Fru1,6bisP concentration and fitting yields an apparent *K*_D_ (*K*_Dapp_) of 9.3 ± 0.3 mM (Fig. 7b), which is in accordance with physiological concentrations of Fru1,6bisP in both mammalian and *S. cerevisiae* cells^41–43^. As a control we also tested the effect of addition of glucose, Fru6P, DHAP and GAP. Glucose had no effect on the H-Ras/Sos1 complex, while Fru6P and DHAP produced a very small effect at non-physiological concentrations (*K*_Dapp_ = 96 ± 40 mM and *K*_Dapp_ = 120 ± 41 mM for Fru6P and DHAP, respectively; Supplementary Fig. 11). Addition of GAP had no effect on the Ras/Sos1 complex up to a concentration of 2 mM, but it led to a very fast decrease in signal due to unspecific dissociation of H-Ras from the biosensor at higher concentrations.

We also performed additional biolayer interferometry (BLI) measurements monitoring the disruption of the Sos1/H-Ras complex by different intermediates of glycolysis and GTP (Supplementary Fig. 11). This showed that only Fru1,6bisP and to a lesser extent Fru6P enhanced the rate of Sos1 dissociation.

### Fru1,6bisP directly binds to human Sos1

To find out whether the observed disruption of the H-Ras/Sos1 complex is due to direct binding of Fru1,6bisP to either H-Ras or Sos1, we determined the thermal stability (melting temperature, *T*_m_) of both proteins in the presence of increasing amounts of Fru1,6bisP, using thermal shift assays^44^. While the thermal stability of H-Ras was quasi unaffected by addition of Fru1,6bisP, the thermal stability of Sos1 increased in the presence of Fru1,6bisP in a dose-dependent way indicating direct binding of Fru1,6bisP to Sos1 (Fig. 7c). Fitting the *T*_m_ value vs. the Fru1,6bisP concentration on a Langmuir-binding model yielded a *K*_D_ of 17.5 ± 2.2 mM. It should be noted that the latter *K*_D_ value corresponds to the real thermodynamic *K*_D_ for binding of Fru1,6bisP to Sos1, while the *K*_Dapp_ for dissociation of the H-Ras/Sos1 complex also depends on kinetic parameters (rate of complex dissociation).

### Glucose activates Ras/MEK/ERK pathway in human cell lines

**Fig. 8:**
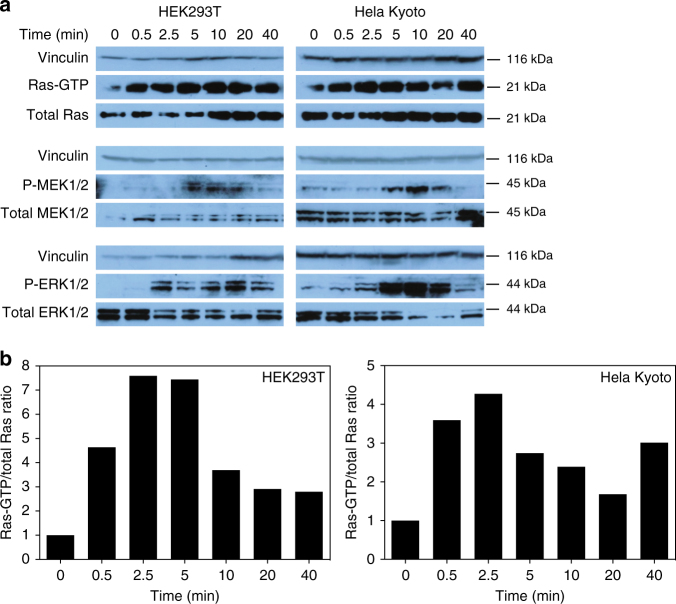
Addition of glucose triggers activation of the Ras/MEK/ERK pathway in glucose-starved human HEK293T and Hela Kyoto cells. **a** Western blot analysis of Ras-GTP, P-MEK and P-ERK levels at different time points after glucose addition to HEK293T (left) and Hela Kyoto (right) cells. Samples at time point 0 were taken prior to glucose addition. Results are representative for at least three repetitions. **b** Quantification of Ras-GTP levels relative to total Ras signals, as depicted in **a**. All values are expressed relative to that of sample *t* = 0, after correction with the vinculin loading control

To assess whether the observed effect of Fru1,6bisP retains physiological relevance in human cells, we studied Ras, MEK, and ERK activation after the addition of glucose to glucose-starved HEK293T and Hela Kyoto cell lines. A 48 h glucose starvation period lowers the glycolytic flux drastically and thus also the Fru1,6bisP level^41^. Re-addition of glucose is known to elevate the intracellular concentration of Fru1,6bisP when glycolysis starts up. The results showed that glucose addition transiently activates Ras, as well as its downstream targets MEK and ERK, both in regular human cells and in cancer cells (Fig. 8a). The ratio of Ras-GTP over total Ras was quantified for better interpretation (Fig. 8b).

### Absence of correlation with proliferation rate

We have also performed multiple experiments to evaluate whether there might be a direct relationship between the proliferation rate of yeast cells, the concentration of Fru1,6bisP and the Ras-GTP level. First, we have measured these three parameters using a series of carbon sources sustaining different proliferation rates. Although there was a tendency for the Ras-GTP level to be lower with lower growth rate, it was certainly not in proportion with the drastically reduced Fru1,6bisP level during growth on non-fermentable carbon sources compared to growth on fermentable sugars (Supplementary Fig. 5). Next, we starved yeast cells for nitrogen on a glucose-containing medium for 50 h and then re-initiated growth by adding a nitrogen source again. This resulted in a drastic drop in Fru1,6bisP and rapid recovery, respectively. However, there was no concomitant change in the level of Ras-GTP at all in the two conditions (Supplementary Fig. 6). We made also use of a yeast strain lacking the *PFK26* and *PFK27* genes, which encode phosphofructokinase 2^45^. It is responsible for the synthesis of Fru2,6bisP, a potent allosteric activator of phosphofructokinase 1. The double-deletion strain had a strongly reduced level of Fru1,6bisP and a slight reduction in growth rate. However, its Ras-GTP level remained unchanged (Supplementary Fig. 7). Next, we investigated whether the slight reduction in growth rate of the *pfk26∆ pfk27∆* strain could be suppressed by an increase in the activity of Ras. The latter was accomplished by single or double deletion of the *IRA1* and *IRA2* genes^37^ (Supplementary Fig. 8) or by expression of *RAS2* ^*val19*^ [10] (Supplementary Fig. 9). However, in none of the two cases was there any increase in the growth rate, neither on glucose nor on galactose medium. All these results indicate that there is no direct relationship between the Fru1,6bisP level and the Ras-GTP level in yeast cells with different proliferation rates. The absence of a clear correlation between Fru1,6bisP and Ras-GTP levels implies that other mechanisms, such as regulation of Ira1,2 activity, must also control the Ras-GTP level. Since yeast strains with constitutively high-Ras activity always showed a tendency for slower growth rates, we overexpressed *RAS2* ^*val19*^ with a multi-copy vector in different yeast strains. In all cases this led to a dramatic reduction in growth rate on glucose medium (Supplementary Fig. 10). This shows that *RAS2* ^*val19*^ is not only toxic under poor growth conditions and in stationary phase, but that very-high expression is also toxic under optimal growth conditions for yeast.

## Discussion

It has been reported that Ras activation stimulates glycolytic rate in tumor cells^46, 47^. The opposite signaling effect that we describe here, i.e., activation of Ras by high glycolytic rate through Fru1,6bisP, may close a reciprocal stimulatory signaling loop between cell proliferation and glycolysis. In this regard, increased cytosolic concentrations of Fru1,6bisP have been found in cancer cells^42, 43^. Moreover, occurrence of mitochondrial dysfunction, accumulation of ROS and switch from respiration to increased glycolytic activity has also been observed in a mammalian cell system in which the K-Ras(G12V) oncogene was expressed from an inducible promoter^48^. Hence, the reciprocal activation between glycolytic flux and Ras may lock cancer cells in a vicious cycle causing both persistent stimulation of cell proliferation and continued maintenance of overactive glycolysis. This would explain the close correlation between the proliferation rate and aggressive character of cancer cells and their fermentation hyperactivity^3, 4, 49^. The new regulatory mechanism is consistent with the synthesis of Fru1,6bisP by phosphofructokinase being a major target of metabolic control pathways, oncogene products and tumor suppressor genes in mammalian cells^22, 46, 47^. It also explains the observation that fructose-1,6-bisphosphatase counteracts carcinoma progression^50^ and that nucleophosmin (NPM1) promotes tumor progression by inhibiting fructose-1,6-bisphosphatase^51^. The similarity between yeast and mammalian cells is further underscored by the finding that in both systems translocation of activated Ras to the mitochondria results in loss of respiratory activity and the appearance of apoptotic markers^48, 52^.

Our results show that neither metabolic deregulation, as observed in the *tps1∆* strain with *ras2∆* or *pPDE2* suppressors, nor the presence of hyperactive Ras, as in a yeast strain expressing Ras2^val19^, is by itself enough to trigger apoptosis and absence of growth on glucose. It is apparently the combined presence of metabolic deregulation, or stalled metabolism, and hyperactive Ras that forces the cells into the pathway of programmed cell death. Similar observations have been made with cancer cells in which glucose deprivation triggers apoptosis^47^ and vitamin C treatment kills *RAS*-oncogene expressing tumor cells, but not wild-type *RAS* containing cells, by inhibition of GAPDH^53^. Combined hyperactivation of Ras and inhibition of glycolysis at the level of GAPDH is also observed in the yeast *tps1∆* mutant, in which it is responsible for induction of apoptosis. This combined requirement may help to explain why *RAS* oncogenes trigger apoptosis only under specific conditions which lead to metabolic deregulation. Another similar mechanism has recently been reported in which Fru1,6bisP directly binds and activates epidermal growth factor receptor (EGFR) causing increased lactate excretion, and EGF signaling activates glycolysis upstream while inhibiting downstream, causing accumulation of intermediates like Fru1,6bisP^54^.

The Biolayer interferometry (BLI) measurements clearly indicate that Fru1,6bisP increases the rate of dissociation of the nucleotide-free Ras-SOS complex. This was unexpected in view of the activating effect of Fru1,6bisP on Ras. Moreover, our results also show that Fru1,6bisP directly binds to SOS1 (and not to Ras). From our results it is hard to pinpoint how exactly this leads to increased activation of Ras in the complex surroundings of the cell. However, it is known that activation of Ras by SOS normally occurs in two steps, where in a first step binding of SOS to GDP-bound Ras leads to dissociation of GDP and in a second step binding of GTP to the nucleotide-free Ras-SOS complex leads to dissociation of the complex concomitant with Ras-GTP loading^40^. Increased activation of Ras in a cellular context could potentially occur through increasing the rate of either step. The observed Fru1,6-bisP-induced dissociation of the nucleotide-free Sos–Ras complex might accelerate activation of Ras by acting between these two steps or through another yet to be identified mechanism. Moreover, at least in yeast, the number of Cdc25 molecules is very low compared to the number of Ras molecules^55^. Hence, a second way in which faster dissociation of Cdc25 and Ras may stimulate Ras activity, is that it may make the limited number of Cdc25 molecules more rapidly available for activation of other Ras molecules.

Since Fru1,6bisP is able to chelate Mg^2+^ ^56^, we have considered the possibility that a reduction in the Mg^2+^ concentration may activate nucleotide exchange on Ras. However, the effect of Fru1,6bisP in the in vitro biophysical experiments is already observed at concentrations much lower than the 10 mM MgCl_2_ used in the buffer. Also the direct binding of Fru1,6bisP on SOS with a *K*_D_ in the same range as the Fru1,6bisP concentration in vivo and the apparent *K*_D_ for Ras-SOS complex dissociation argues against a mere effect of Fru1,6bisP as a chelator.

We have also performed multiple experiments to evaluate whether there might be a direct relationship between the proliferation rate of yeast cells, the concentration of Fru1,6bisP and the Ras-GTP level. However, no clear correlation could be found (see Supplementary Figs. 5–10). Hence, although both yeast and mammalian cells show within min rapid glucose-induced activation of Ras and its downstream targets, other mechanisms may intervene in hierarchical control of the cell proliferation rate.

In conclusion, we have identified a direct regulatory connection between Fru1,6bisP in glycolysis and the Ras proteins, that seems to be evolutionary conserved in yeast and mammalian cells. It is responsible for induction of apoptosis in yeast cells with overactive influx of glucose into glycolysis and stalled flux at the level of GAPDH. We suggest that Fru1,6bisP activation of Ras constitutes a key mechanism through which the Warburg effect might stimulate oncogenic potency.

## Methods

### Yeast strains and plasmids

All *S. cerevisiae* strains used in this work are listed in Supplementary Table 1. They all share the same genetic background, i.e. W303. The YEplac195 expression vector contains the promoter and terminator sequences of the 5′ and 3′ regions of the *PDE2* gene. The YCp*Ras2* ^*val19*^ plasmid contains promoter and terminator sequences of the 5′ and 3′ regions of the *RAS2* ^*val19*^ gene.

### Growth conditions

When no selection was required, yeast cells were grown in rich YP (1% (w/v) yeast extract, 2% (w/v) bactopeptone) medium, supplemented with 100 mg/L adenine and either 100 mM glucose, 100 mM galactose, 220 mM ethanol or 325 mM glycerol. In case of selection for the presence of an auxotrophic marker either on a plasmid or in the genome, cells were grown in synthetic medium containing 0.17% (w/v) yeast nitrogen base without amino acids, 0.5% (w/v) ammonium sulfate, the appropriate amount of the required synthetic ‘drop out’ amino acid and nucleotide mixture and supplemented with 100 mM glucose, 100 mM galactose, 220 mM ethanol or 325 mM glycerol. The pH was adjusted with 4 M KOH to 5.5 for liquid medium and 6.5 for solid medium. For solid medium, 1.75% (w/v) agar was added after pH adjustment. Strains were grown at 30 °C. Liquid cultures were grown under continuous shaking (180 r.p.m.).

### Expression and purification of RBD-GST from *Escherichia coli*

Ras-binding domain of the Raf1-kinase (RBD) GST-fusion protein was constructed in the pGEX2T-1 expression vector and expressed in the *E. coli* BL21 strain. Cells were grown for 1 h at 37 °C prior to addition of IPTG to a final concentration of 0.3 mM to induce gene expression from the pGEX2T-1 plasmid. Induction lasted for 3 h at 30 °C. Cells were harvested and washed with ice-cold PBS buffer. Cells were resuspended in 5 mL per L culture lysis buffer containing 50 mM Tris-HCl pH 7.5, 50 mM NaCl, 2 mM EDTA pH 8, 1 mM EGTA pH 8, 20% sucrose and 5 mg/mL lysozyme at a concentration of 10 mL per L of culture and incubated for at least 15 min. 3 volumes of ice-cold buffer containing 50 mM Tris-HCl pH 7.5, 150 mM NaCl, 1% Triton X-100 and protease inhibitor cocktail (complete, EDTA-free, Roche) was added. Cells were lysed by sonicating three times for 15 s. Lysates were clarified by centrifugation at 20,000 r.p.m. for 25 min at 12,000 × *g*. The resulting supernatant fraction was incubated with 2 mL per L of initial culture of a 50–50 slurry of glutathione-sepharose beads (GE Healthcare) prewashed with 1 x TBST (50 mM Tris-HCl pH 7.5, 150 mM NaCl, 1% Triton X-100 and 1 mM DTT) and incubated for 1 h in a roller drum at 4 °C. Beads were collected by centrifugation at 1800 rpm for 1 min and washed 4 times with TBST. Beads were resuspended in TBS (TBST minus Triton) at a final concentration of 10%. The addition of 0.02% NaN_3_ allowed keeping this solution for up to 6 months.

### Preparation of a yeast crude cell extract

Cells were grown to an OD of 3–4 and collected on a microfilter connected to a vacuum pump. After washing with ice-cold water, 200 mg cells were collected in a screw cap tube. 0.2 g of glass beads were added together with 500 µL of lysis buffer containing 50 mM Tris-HCl pH 7.5, 10% glycerol, 2.5 mM MgCl_2_, 1% Igepal (NP-40) and 200 mM NaCl. The cells were broken by shaking three times 20 s in a fast prep apparatus. A clarified crude protein extract was obtained after 5 min centrifugation at 8000 rpm. Protein concentration was measured by mixing 20 µL of sample together with 300 µL of the Pierce 660 nm protein measurement buffer and absorbance was measured at 660 nm using a bovine serum albumin (BSA) standard curve.

### Preparation of permeabilized spheroplasts

The protocol for preparation of spheroplasts was adapted from previous reports^35, 57^. Strains were grown in 1 L YP galactose to exponential phase (OD_600_ = 2). The cells were washed with water and resuspended at a concentration of 200 mg/mL in digestion buffer containing 1.2 M sorbitol, 60 mM potassium phosphate buffer pH 7.5 (K_2_HPO_4_ + KH_2_PO_4_), 1 mM EDTA pH 8, 10 µL/mL β-mercaptoethanol and 100 units/mL lyticase. This cell suspension was incubated at 30 °C and digestion of the cell walls was followed by the decrease in OD_600_ for a sample of the spheroplast suspension was treated with 5% SDS (10 µL spheroplast suspension + 990 µL SDS). When OD_600_ had dropped with 80% in this assay, the spheroplasts were considered appropriate for use and the lyticase digestion was arrested by addition of four volumes of ice-cold 1.2 M sorbitol solution. Spheroplasts were washed two times with ice-cold 1.2 M sorbitol and resuspended in spheroplast buffer containing 1.2 M sorbitol, 0.75 mM EDTA, 2 mM MgSO_4_, 1.8 mM NaCl and 10 mM potassium phosphate buffer pH 6.8. A sample was taken of the spheroplast suspension to measure protein concentration. For that purpose, 10 µL spheroplast suspension was diluted with 90 µL of water and the spheroplasts homogenized by vortexing. Subsequently, 20 µL of the resulting solution was mixed with 300 µL of the Pierce protein measurement buffer and the protein concentration was determined by measuring OD at 660 nm. The spheroplasts were then diluted with spheroplast buffer to a protein concentration of 2 mg/mL. To permeabilize the spheroplasts, they were incubated for 10 min at 30 °C with 20 µL/mL nystatin.

### Ras activation assay

Two different Ras activation assays were used. Ras activation was either studied in vivo upon addition of glucose to galactose-grown cells, or in situ, upon addition of different glycolytic intermediates, which cannot be taken up by intact cells, to permeabilized spheroplasts. The level of Ras-GTP was determined with the RBD-GST pull-down assay, as described previously^15^. The responses in the spheroplast preparations showed some quantitative variability, which is likely due to the elaborate spheroplast preparation protocol, but qualitatively the responses were always comparable.

### Ras activation as determined with samples taken in vivo

For the in vivo Ras activation assay, glucose was added to galactose-grown cells and the cells were incubated further for 10 min. Crude cell extracts were prepared and a volume containing 2 mg of total protein was added to the beads bound to RBD-GST and incubated for 1 h at 4 °C on a roller drum. After incubation, the beads were washed three times with the lysis buffer used to make the crude extracts. After washing, the beads were dried by removing all liquid with a narrow orifice 30 g needle. To the dried beads, 20 µL of 2× loading buffer was added. This buffer contains 100 mM Tris-HCl pH 8, 20 mM β-mercaptoethanol, 4% SDS, 0.2% bromophenol-blue and 20% glycerol. This solution was boiled for 5 min at 96 °C. A volume with 20 µg of total protein was used for the control in which the total Ras content was determined and an equal amount of 2× loading dye was added and boiled. The samples were then ready for western blotting.

### Ras activation in permeabilized spheroplasts (in situ)

Glycolytic metabolites and GDP were dissolved in H_2_O to a final concentration of 100 mM, except glyceraldehyde-3-phosphate, which was dissolved in a potassium phosphate buffer (because it is only available as a very acidic liquid solution). GTP was dissolved in H_2_O to a final concentration of 20 mM. 50 µL of the stock solutions was added to 1 mL permeabilized spheroplasts to obtain a final concentration of 5 mM for the metabolites and GDP and a final concentration of 1 mM for GTP. The spheroplasts were incubated for 3 min at 30 °C and after incubation snap frozen in liquid nitrogen. 330 µL of a 4× lysis buffer was added to the frozen spheroplasts. Spheroplast lysis was achieved by simple vortexing and the supernatant was cleared by centrifugation at 8000 rpm for 5 min. Crude extracts were incubated with the RBD-GST beads and further processed as described in the previous section.

### Glucose activation of Ras in mammalian and cancer cells

Mammalian HEK293T cells (ATCC, USA) and Hela Kyoto cancer cells (a kind gift of Dr. D. Gerlich^58^), characterized by Short Tandem Repeat profiling, were used at low passage number (<15) immediately after receipt or after resuscitation from early stocks. Mycoplasma contamination was checked bimonthly with Venor Gem Classic kit (Minerva Biolabs). Both cell lines were grown in Dulbecco’s Modified Eagle Medium (DMEM) medium without glucose (11966-025, Invitrogen) supplemented with 2 mM glucose (Life Technologies), 20 mM galactose (Sigma), 6 mM l-glutamine (Sigma), 1 mM sodium pyruvate (Invitrogen), 10% Fetal Calf Serum (FCS, GE Healthcare), 5 mM HEPES buffer (pH 7.2–7.5) (Thermo Fischer Scientific), 100 units/mL penicillin (Sigma) and 100 mg/mL streptomycin (Sigma), prior to a 48 h glucose starvation period in the same medium without glucose supplementation. Activation of Ras, MEK and ERK was monitored in cells incubated in DMEM medium without glucose (D5030, Sigma-Aldrich) after supplementation with 20 mM glucose, 5 mM HEPES buffer (pH 7.2–7.5), 10% FCS, 100 units/mL penicillin and 100 mg/mL streptomycin, for different time periods (0.5, 2.5, 5, 10, 20 and 40 min). Before lysis, cells were washed with ice-cold PBS (Sigma) and collected in lysis buffer (1% NP40, 50 mM Tris-HCl (pH 7.4), 150 mM NaCl, 0.5 mM EDTA, 5 mM MgCl_2_ and protease/phosphatase inhibitor cocktails (Roche)). Lysates were centrifuged (14,000 × *g*, 15 min, 4 °C) and the supernatants were prepared for western blot analysis. Active Ras-GTP levels were assessed following pull-down using RBD-GST-fusion protein beads (1 h, 4 °C) (see above).

### Western blotting

All samples were boiled for 5 min at 96 °C and briefly centrifuged before loading on the electrophoresis gel. Proteins were separated via SDS-PAGE (NuPAGE 4–12% Bis-Tris gel, Invitrogen) in NuPAGE MOPS SDS running buffer (Invitrogen) at a constant voltage of 150–200 V. After electrophoresis, proteins were transferred onto nitrocellulose membranes (HybondC extra, GE Healthcare) by blotting for 60 min at 400 mA in blotting buffer (NuPAGE MOPS SDS running buffer, with 20% (v/v) methanol). Aspecific antibody binding was prevented by incubating the membrane in blocking buffer ((5% (w/v) BSA in TBS-Tween buffer (25 mM Tris-HCl pH 8, 150 mM NaCl, 0.05% (v/v) Tween-20)) for 1 h at room temperature. Subsequently, the blots were incubated overnight at 4 °C in blocking buffer with the appropriate antibody: yeast Ras2 (Sc-6759, Santa Cruz, 1:1000, goat pAb), Human Ras (anti-Pan-Ras, Calbiochem, OP40-100UG, 1:500, mouse mAb), ERK (anti-p44/42 MAPK (ERK1/2), Cell Signaling Technology, #9102, 1:1000, rabbit pAb), P-ERK (anti-phospho-p44/42 MAPK (ERK1/2) (T202/Y204), Cell Signaling Technology, #9101S, 1:1000, rabbit pAb), MEK (anti-MEK1/2 (L38C12), Cell Signaling Technology, #4694, 1:1000, mouse mAb, P-MEK (anti-phospho-MEK1/2 (S217/221) (41G9), Cell Signaling Technology, #9154S, 1:1000, rabbit), Vinculin (anti-vinculin, Sigma-Aldrich, V9131, 1:10000, mouse mAb). Subsequently, the blots were washed three times with TBS-Tween buffer and incubated with the appropriate secondary antibody (Sc-2020, Santa Cruz) in TBS-Tween. After washing three times with TBS-Tween, the membranes were incubated with Supersignal chemiluminescence substrate (Pierce). Proteins were visualized by exposure of the membrane in the LAS4000 mini digital system (Fujifilm). Signals were quantified with Aida software. Uncropped scans of key western blots presented in the main figures are provided in Supplementary Fig. 12.

### Metabolite determinations

The cells for the metabolite measurements were obtained by culturing in Complete Synthetic medium with 100 mM galactose to an OD of 2–3. They were harvested by centrifugation and washed two times with 25 mM β-morpholinoethanesulfonic acid (MES) pH 6. After the last wash step, the cells were collected on a filter and weighed. For short-term (up to 10 min) measurement of the intracellular concentration of metabolites, the cells were then suspended at a concentration of 75 mg wet weight/mL in 25 mM MES buffer and incubated in a waterbath at 30 °C. Samples were taken by quenching 2 mL of cell suspension in 10 mL of 60% methanol at—40 °C^59^. For metabolite determination after glucose addition, a final concentration of 100 mM glucose was added to the cells and samples were taken as a function of time by taking 2.5 mL cell suspension which was quenched in ice-cold methanol. The cells were centrifuged at −20 °C, at 3000 rpm for 3 min. The supernatant was removed completely by the use of a vacuum pump and resuspended in 500 µL of 1 M perchloric acid (HClO_4_). The cells were transferred to a screw cap tube and broken with glass beads in the fast prep apparatus (two times 20 s at speed setting of 6). 500 µL of 1 M HClO_4_ was added again and the mixture spun down for 4 min at 12,000 r.p.m., at 4 °C. 250 µL was mixed with 10 µL thymol blue (25 mg/100 mL) and 50 µL of 5 M K_2_CO_3_. The tubes were left open for at least 15 min to make sure all CO_2_ was released. 200 µL was transferred to a new test tube and mixed with 100 µL of 1 M HCl and 10 µL of 2 M Tris-HCl pH 7.5.

For long-term (up to 30 h) measurement of the intracellular concentration of different metabolites, cells were also grown in Complete Synthetic medium with 100 mM galactose to an OD of 2–3, after which 100 mM glucose was added in the cultures. Samples were taken as a function of time as described for the short-term experiments. At the same times a sample with equal volume was collected by filtration to determine the cell dry weight.

### Glucose-6-phosphate and ATP

50 µL of the sample was mixed with 150 µL of reaction buffer containing 100 mM Hepes pH 7.6, 0.8 mg/mL NADP and 10 mM MgCl_2_. To measure glucose-6-phosphate the enzyme glucose-6-phosphate dehydrogenase (G6PDH) was added in a concentration of 50 µg/mL. The NADPH formed in this reaction was measured at a wavelength of 340 nm. The amount of NADPH determined is directly proportional to the amount of glucose-6-phosphate present in the sample. For ATP determination, hexokinase at a concentration of 100 µg/mL was added together with 0.5 mM glucose. Hexokinase will produce glucose-6-phosphate with the glucose added and the ATP present in the sample and the glucose-6-phosphate will be converted again by G6PDH with the generation of NADPH, that is measured and is directly proportional to the ATP concentration present in the sample.

### Fructose-1,6-bisphosphate

50 µL of the sample was mixed with 150 µL of reaction buffer containing 100 mM Hepes pH 7.6 and 0.3 mM NADH, 2.5 µg/mL glyceraldehyde-3-phosphate dehydrogenase and 2.5 µg/mL triose phosphate isomerase. For measurement of fructose-1,6-bisphosphate, aldolase was added at a concentration of 100 µg/mL and the consumption of NADH was measured at a wavelength of 340 nm.

### Reactive oxygen species

The production of reactive oxygen species (ROS) was assessed using dihydrorhodamine 123 (DHR; Sigma-Aldrich) as described previously^60^. Cells were grown in YP galactose medium until mid exponential phase, a sample was collected and 100 mM glucose was added after which a new sample was taken. 5 µg/mL DHR was added to the cells and incubated for 2 h. ROS production was visualized using fluorescence microscopy (Axioplan 2 imaging, Zeiss).

### Annexin staining of phosphatidylserine exposure

Cells were stained using the Annexin-V-FLUOS Staining Kit (Roche) as described previously^60^. After growing cells to mid exponential phase in YP galactose medium, 100 mM glucose was added. After 16 h the cells were collected, washed with PBS buffer and centrifuged for 5 min at 200 × *g*. Annexin-V-FLUOS labeling solution was prepared according to the instructions of the manufacturer: 1 mL of incubation buffer and 20 µL of Annexin-V-FLUOS labeling reagent and 10 µL of propidium iodide solution. Washed cells were incubated in 100 µL of this labeling solution for 15 min after which the cells were observed in the fluorescence microscope. Annexin-V-FLUOS excitation was performed at 488 nm and emission was measured at 518 nm. Propidium iodide excitation was performed at 488–540 nm and emission was measured at 617 nm.

### Isolation of mitochondria for cytochrome c release

The procedure was adapted from a previous report^61^. A 1 L culture was used for each sample. 100 mM glucose was added to a 1 L culture after which the culture was incubated for the indicated time. The cells were harvested by centrifugation at 3000 rpm for 10 min and washed two times with 1.2 M sorbitol. The cells were then resuspended in 5 mL of digestion buffer per g cells. The digestion buffer contained 1.2 M sorbitol, 60 mM potassium phosphate buffer pH 7.5, 1 mM EDTA pH 8, 1% β-mercaptoethanol (w/v) and 1% zymolyase 20 T. The mixture was incubated for 1 h at 30 °C. The spheroplasts formed were collected by centrifugation for 5 min at 1500 × *g*, washed two additional times with 1.2 M sorbitol and resuspended in 3 mL of ST-Buffer, containing 0.25 M sorbitol, 20 mM Tris-HCl pH 7.5, 1 mM EDTA and protease inhibitor cocktail (Roche), per g of cells. The suspension was incubated with agitation at 4 °C until the spheroplasts were osmotically broken. Cell debris were removed by centrifugation at 2000 × *g* for 5 min and the supernatant was again centrifuged at 12,000 × *g* for 15 min to precipitate mitochondria. Both the post-mitochondrial supernatant (SN) and the pellet (the mitochondrial pellet was resuspended in 0.5 M sorbitol) were used to detect cytochrome c by Western blot analysis as described previously^62^.

### Purification of H-Ras and Sos1 (residues 564–1049)

The open reading frames, codon optimized for expression in *E. coli*, for human H-Ras (residues 1–166) and Sos1 (residues 564–1049) were ordered from Genscript, Inc., and cloned into the pET28b vector using the NdeI and XhoI restriction sites (Novagen). His-tagged H-Ras and Sos1 proteins were expressed in *E. coli* BL21 and purified by immobilized metal ion affinity chromatography (IMAC) and size-exclusion chromatography as described previously^40^. The His-tag was either kept or cleaved off by TEV protease (12 h at 4 °C), after which the uncleaved protein was removed by a second IMAC.

### Biolayer Interferometry (BLI) experiments

Biolayer interferometry measurements were performed using an Octet Red96 (Forte Bio, Inc.) system at 25 °C, shaking at 1000 rpm and in a buffer containing 25 mM Hepes pH 7.5, 0.15 M NaCl and 0.01 M MgCl_2_. To monitor disruption of the H-Ras/Sos1 complex by Fru1,6bisP, in a first step the Ni^2+^-coated biosensors were equilibrated in buffer and a baseline was collected for 30 s. Subsequently the biosensors were loaded with his-tagged H-Ras at a concentration of 12.5 µg/mL. After another baseline acquisition (200 s) Sos1 (residues 564–1049) was added at 0.5 µM (200 s). Finally, the biosensors were transferred to wells containing buffer supplemented with increasing concentrations of Fru1,6bisP and dissociation of the H-Ras/Sos1 complex was monitored for 700 s. All samples were prepared and measured in triplicate. To obtain an apparent dissociation constant (dependent on the dissociation kinetics of the complex) the signal 150 s after transfer to Fru1,6bisP was plotted vs. the Fru1,6bisP concentration. The resulting titration curve was fitted on a Langmuir equation. A similar procedure was used to study the effect of Fru6P, DHAP, GAP and glucose on the Ras/Sos1 complex.

### Fluorescence-based thermal shift assay

Thermal shift assays (TSA) were performed with Sos1 (residues 564–1049) and H-Ras in presence of increasing concentrations of Fru1,6bisP. Thermal unfolding was detected by following SYPRO orange fluorescence using a CFX connect Real-Time PCR System (Bio-Rad). 0.2 mg/mL of either protein was combined with 5X SYPRO Orange Protein Gel Stain (Thermo Fisher Scientific) at concentrations of Fru1,6bisP ranging from 0 mM to 100 mM, in a final volume of 20 µl. Fluorescence was measured from 20 °C to 80 °C at a rate of 0.5 °C/30 s. All samples were prepared and measured in triplicate. The melting temperatures *T*_m_ were determined by fitting the first derivatives of the data using Prism with a Gaussian distribution/Boltzmann sigmoidal equation. The *T*_m_ values were plotted vs. the added ligand concentrations and these binding curves were analyzed using Prism and fitted with a Langmuir-binding model.

### Reproducibility of the results

All experiments were repeated at least three times with consistent results.

### Data availability

The authors declare that all data supporting the findings of this study are available within the article and its Supplementary Information files and from the corresponding author upon reasonable request.

## Electronic supplementary material

Supplementary Information
