# Supplementary material for: Fructose-1,6-bisphosphate couples glycolytic flux to activation of Ras
Source: Nat Commun. 2017 Oct 13;8:922. doi: 10.1038/s41467-017-01019-z (PMC5640605; doi:10.1038/s41467-017-01019-z)
Supplement: Supplementary file 1 — Supplementary Information [file 41467_2017_1019_MOESM1_ESM.pdf]

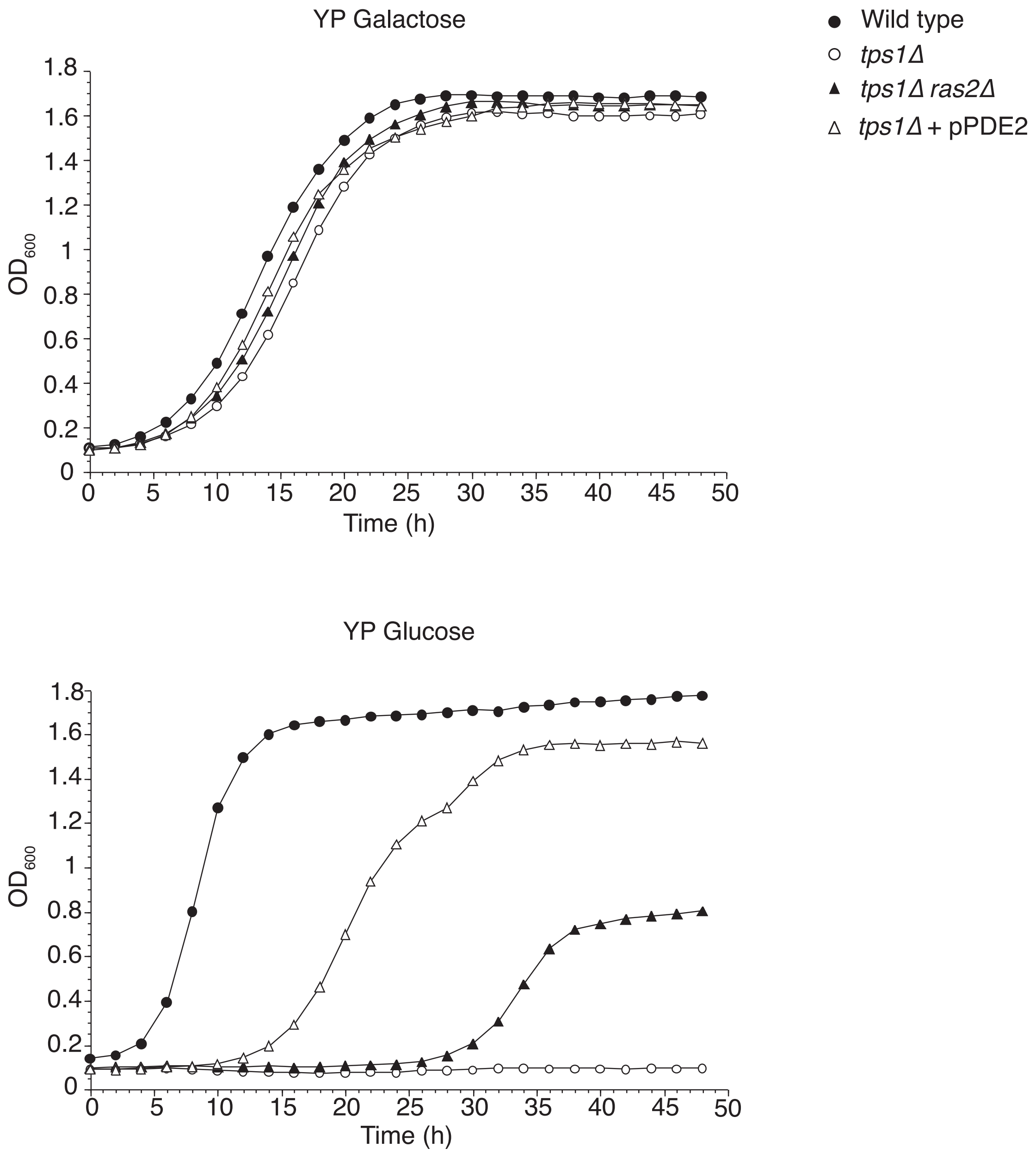

Supplementary Fig. 1

Growth of the *tps1*Δ strain and *tps1*Δ suppressor strains in rich liquid medium with 100 mM galactose or 100 mM glucose.

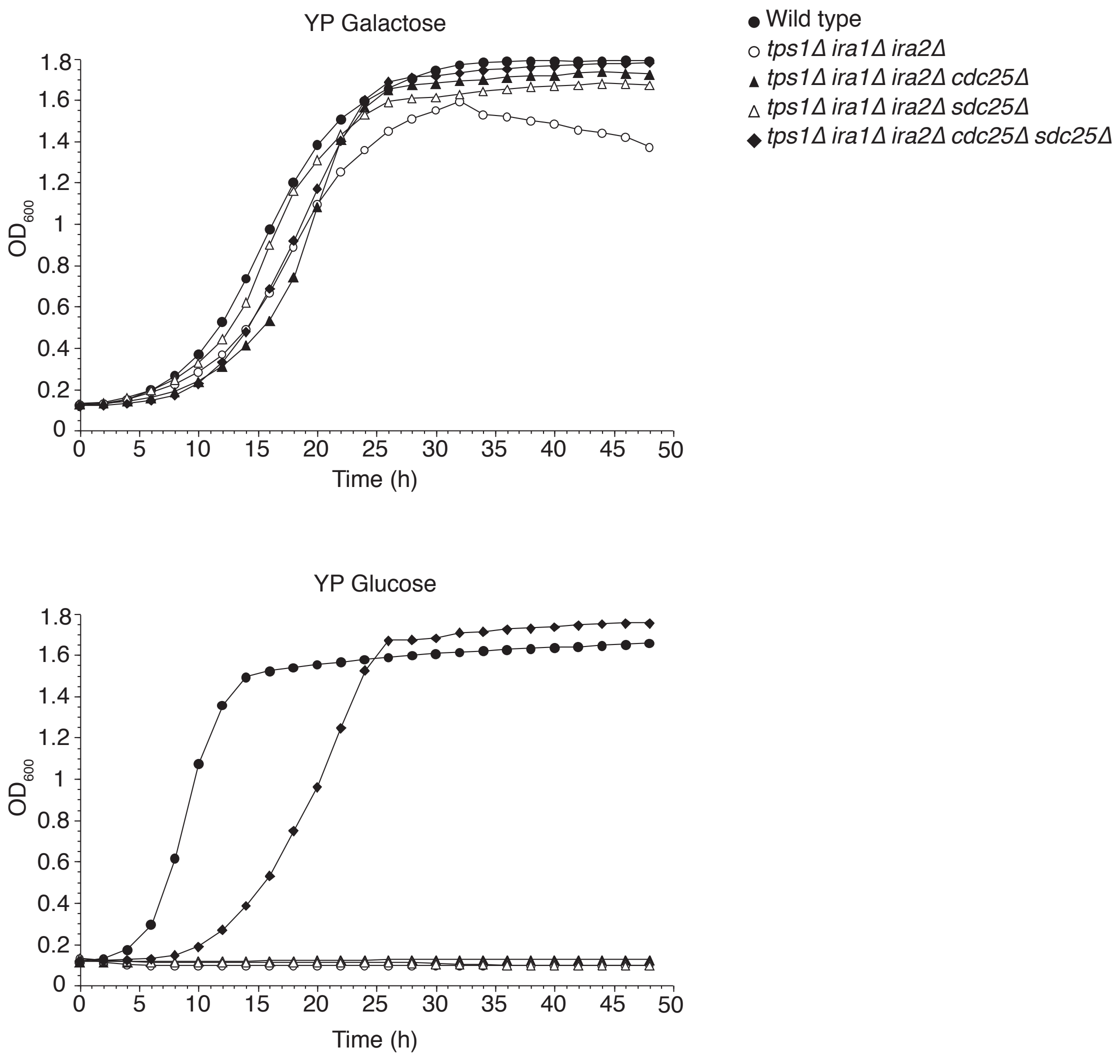

Supplementary Fig. 2

Growth of the *tps1*Δ strain and *tps1*Δ strains with additional deletions in Ras-GEF or Ras-GAP factors, in rich liquid medium with 100 mM galactose or 100 mM glucose.

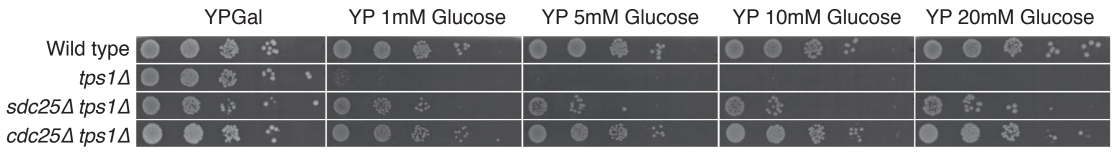

Supplementary Fig. 3

Partial restoration of growth on glucose in the *tps1*Δ strain by deletion of *SDC25* or *CDC25*.

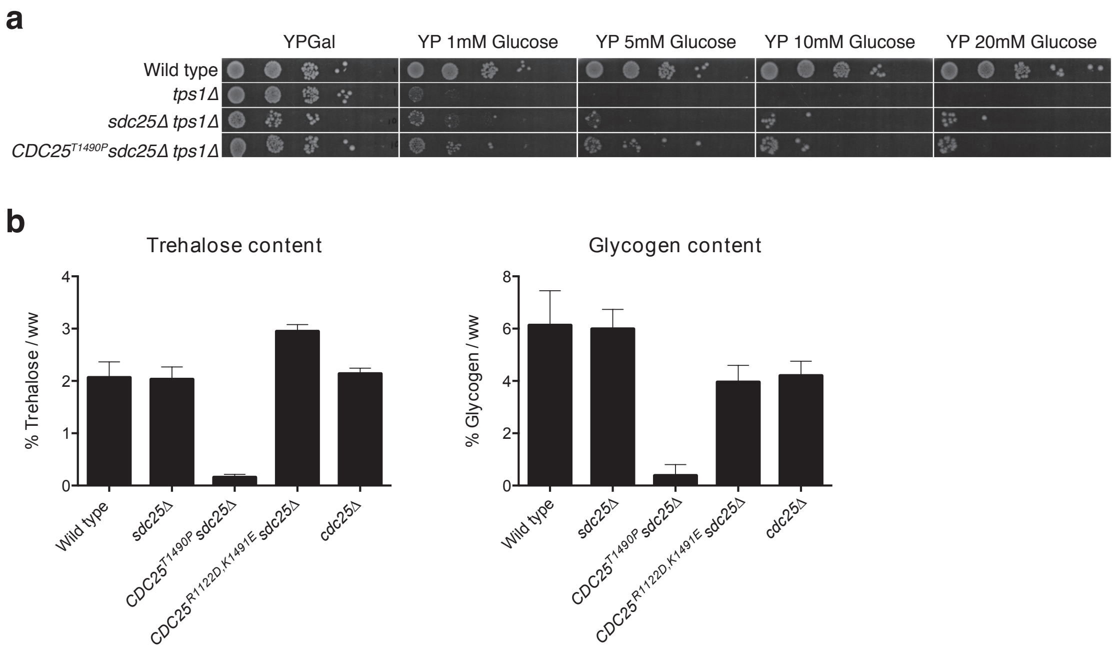

Supplementary Fig. 4

Phenotypes of a *tps1*Δ strain expressing Cdc25^{T1490P} instead of wild type Cdc25. **a,** Absence of recovery of growth on glucose. **b,** Reduced trehalose and glycogen levels in cells grown on glycerol into stationary phase.

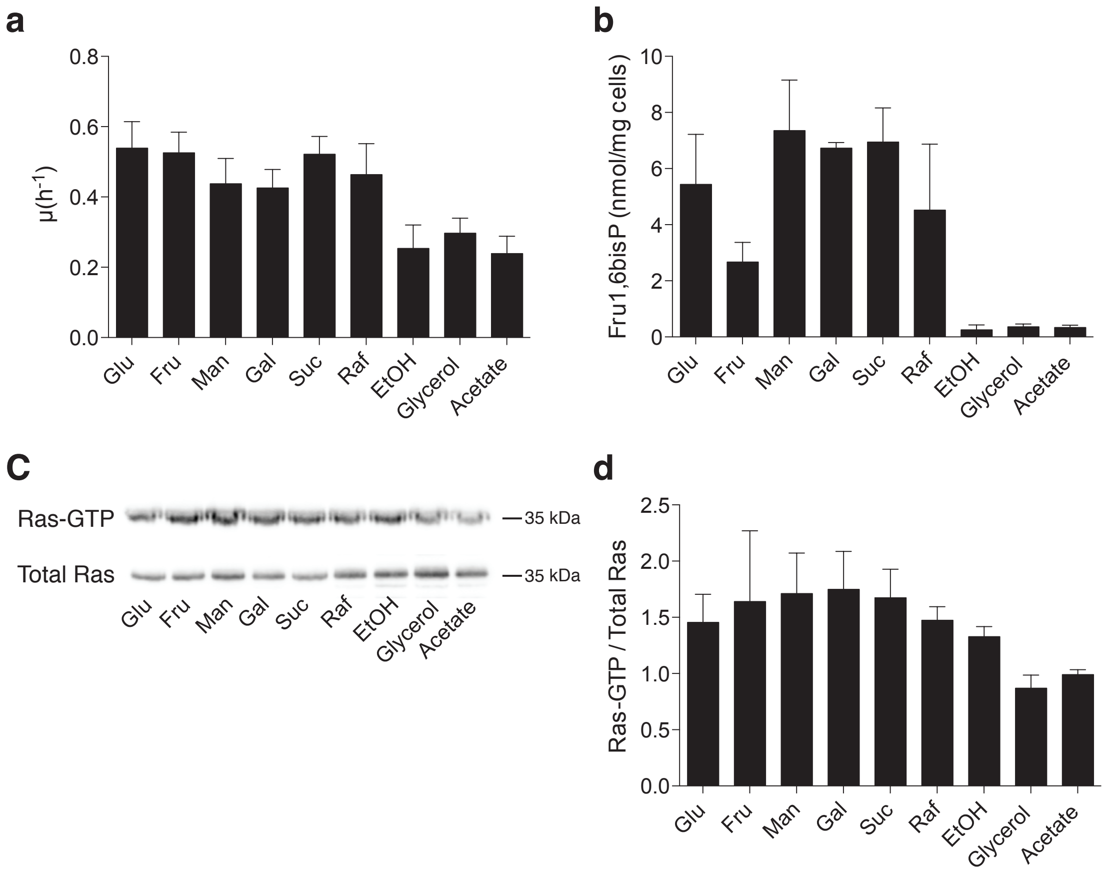

Supplementary Fig. 5

Specific growth rate (**a**) and Fru1,6bisP intracellular pool (**b**) do not show a correlation with the Ras-GTP level (**c,d**) during exponential growth of the wild type strain on rich medium containing different carbon sources (2% w/v or v/v for ethanol and glycerol). Values represent average ± SD of three independent experiments. (**c**) Representative Western blot experiment for determination of the Ras-GTP level. (**d**) The Western blots were quantified and the Ras-GTP level is shown relative to the amount of total Ras.

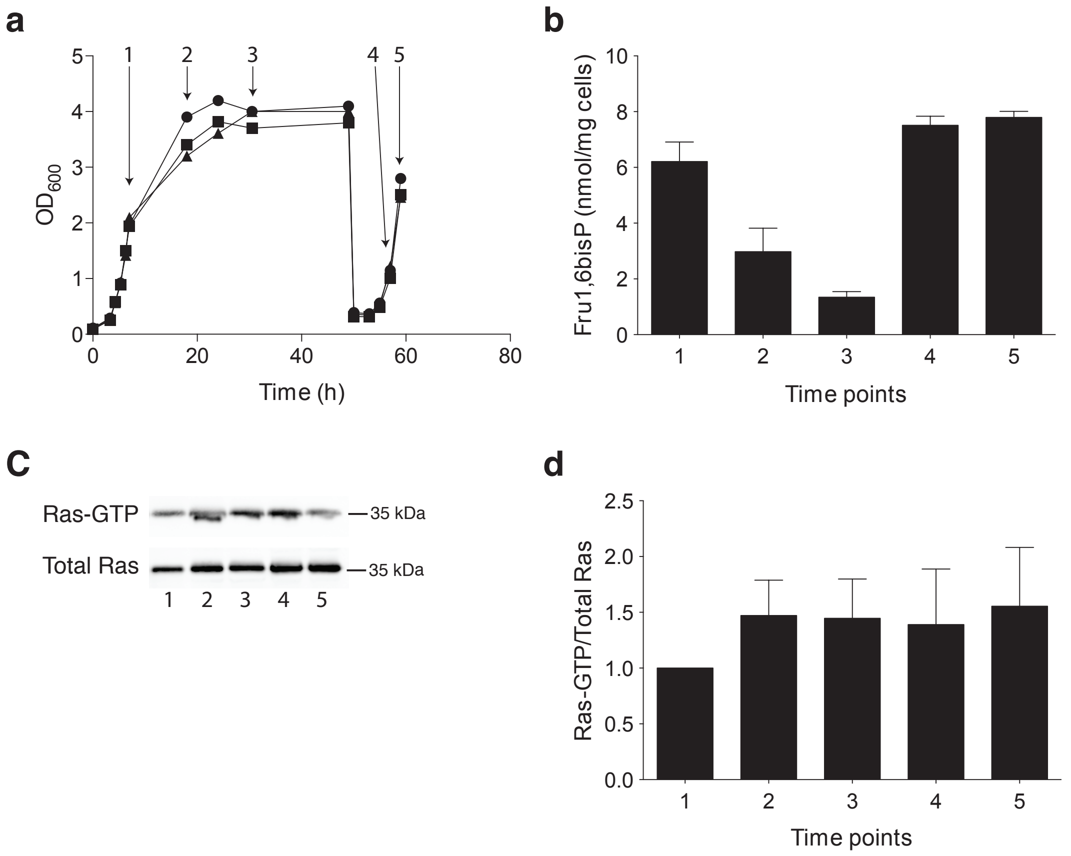

Supplementary Fig. 6

During nitrogen starvation on a glucose-containing medium, the growth rate and the intracellular Fru1,6bisP level drop but the Ras-GTP level remains unchanged. The cells of the wild type strain were grown on complete minimal medium, containing 100 mM glucose and 40 mM ammonium sulfate, till OD = 2, after which they were transferred to glucose-containing nitrogen starvation medium, further incubated up to 48h after the initial inoculation and subsequently resuspended again in the complete minimal medium with 100 mM glucose and 40 mM ammonium sulfate. (**a**) Optical density of three independent cultures. (**b**), Intracellular Fru1,6bisP content. (**c,d**), Ras-GTP level. In (**b**) and (**d**) values represent average ± SD. (**c**) Representative Western blot experiment. (**d**) Quantification of the Western blot experiments. The ratio Ras-GTP/total Ras is expressed relative to the corresponding value at the first sampling time point ('1') for each culture.

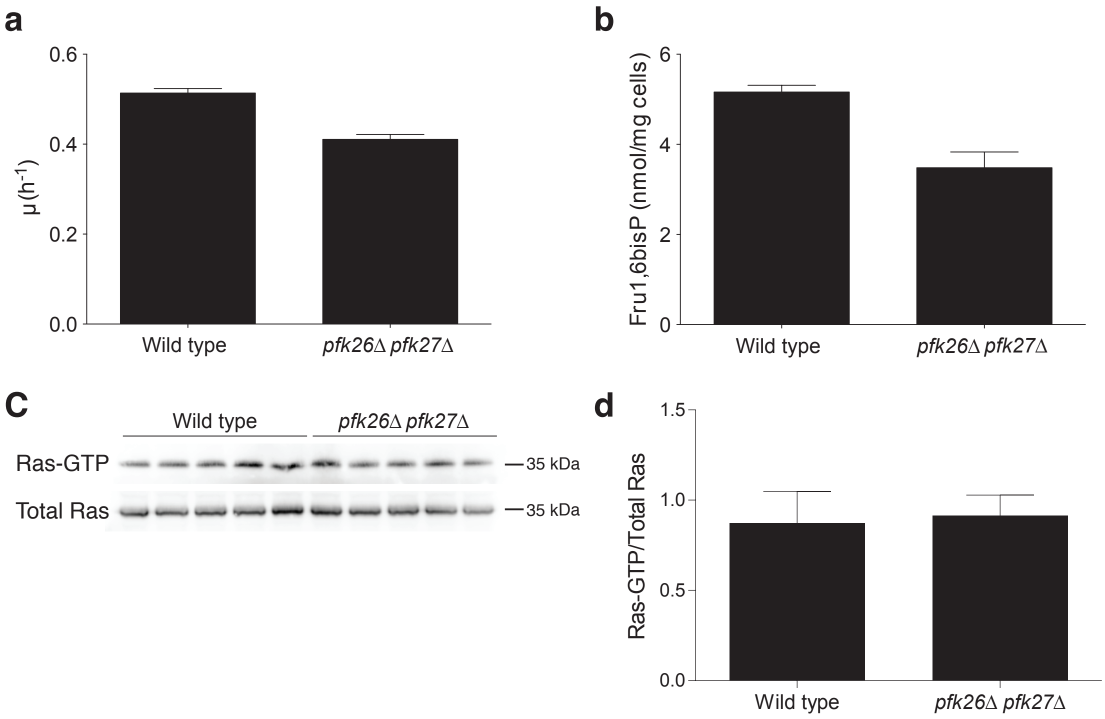

Supplementary Fig. 7

Deletion of the *PFK26* and *PFK27* genes causes a drop in the growth rate (**a**) and intracellular Fru1,6bisP level (**b**) but the Ras-GTP level remains unchanged (**c,d**). Values represent average ± SD of five independent cultures growing exponentially on YPD. The band intensities in (**c**) were quantified and the Ras-GTP level is shown relative to the amount of total Ras in (**d**).

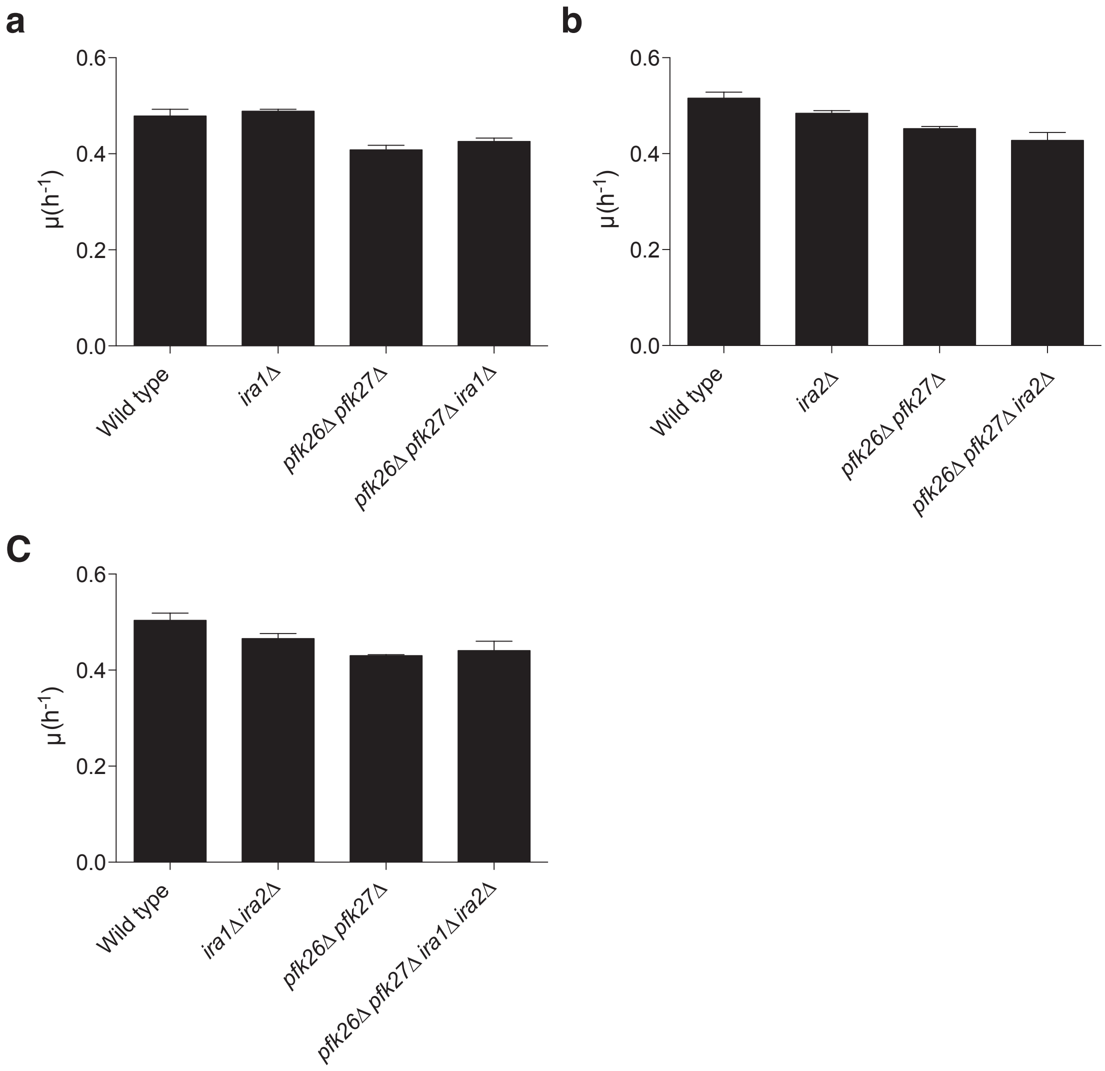

Supplementary Fig. 8

Deletion of *IRA1*(**a**), *IRA2* (**b**) or both *IRA1* and *IRA2* (**c**) does not enhance the growth rate of the *pfk26Δ pfk27Δ* strain. Values represent average ± SD of three independent cultures growing exponentially on YPD.

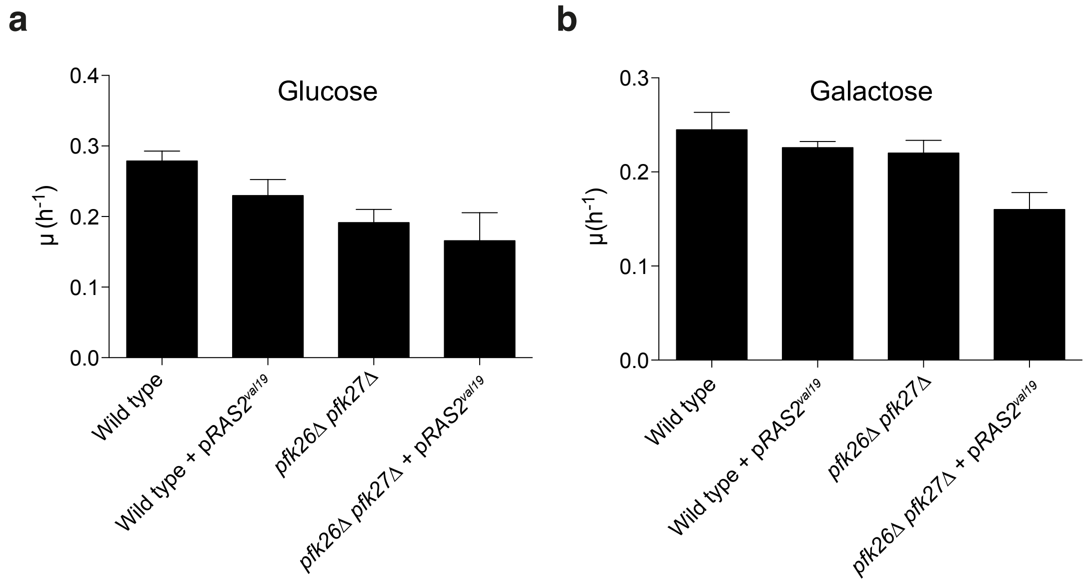

Supplementary Fig. 9

Expression of the constitutively active *RAS2^{val19}* gene does not enhance the growth rate of the *pfk26Δ pfk27Δ* strain. Strains were transformed with the low-copy number plasmid YCplac33 bearing the *RAS2^{val19}* gene or with the empty plasmid. Values represent average ± SD of three independent cultures growing exponentially on minimal medium minus uracil with 100 mM glucose (**a**) or galactose (**b**).

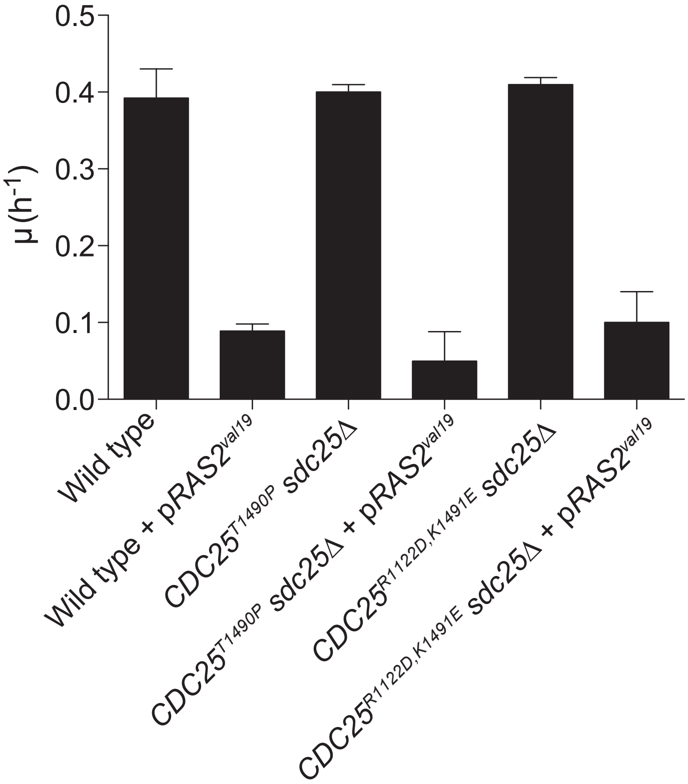

Supplementary Fig. 10

Overexpression of the $RAS2^{val19}$ allele severely reduces the growth rate in different yeast strains. Strains were transformed with the high-copy number plasmid YEplac195 bearing the $RAS2^{val19}$ gene or with the empty plasmid. Values represent average ± SD of three independent cultures growing exponentially on minimal medium minus uracil with 100 mM glucose.

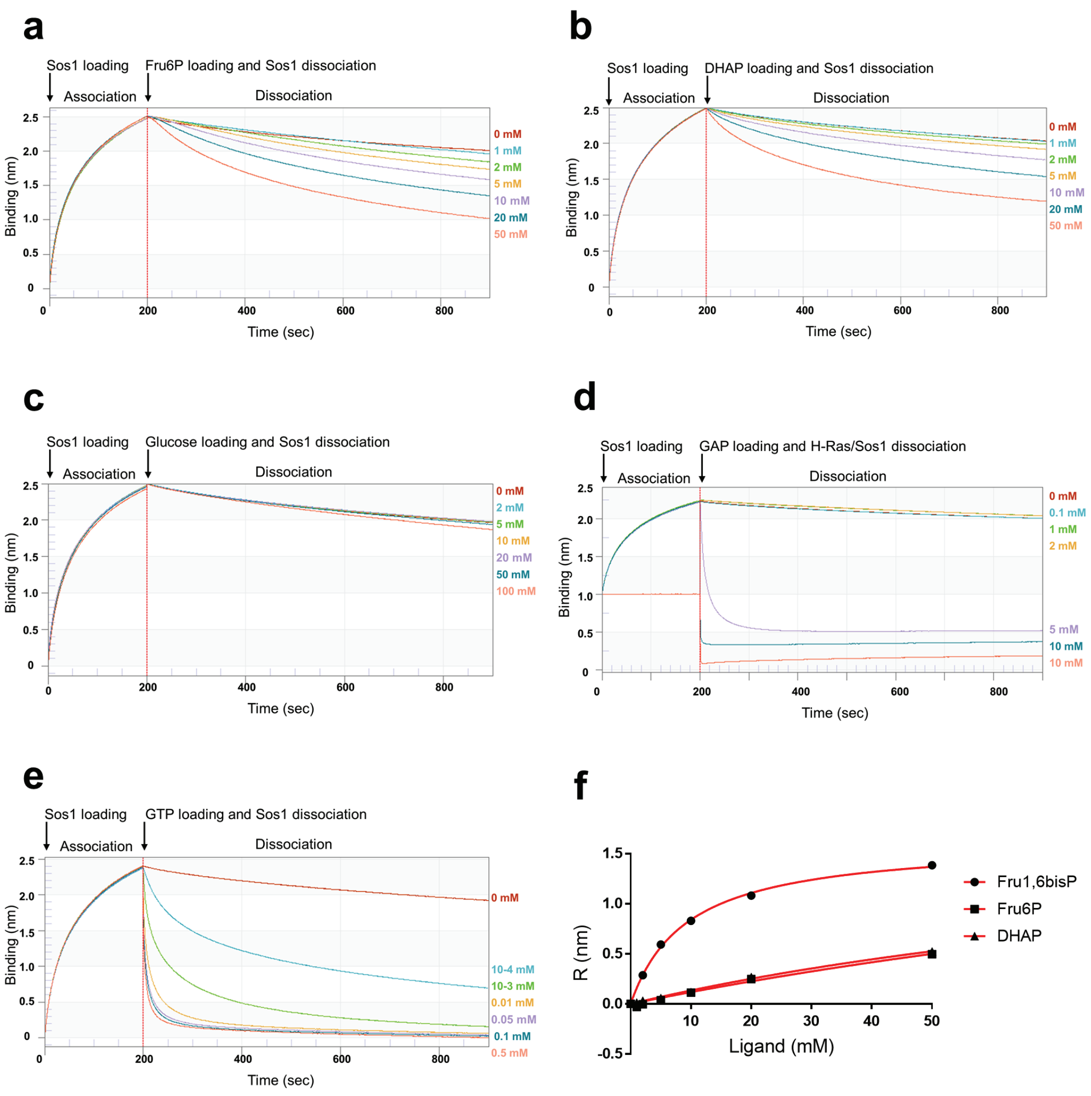

Supplementary Fig. 11

Biolayer interferometry (BLI) measurements monitoring the disruption of the Sos1/H-Ras complex by different intermediates of glycolysis and GTP. His-tagged H-Ras was coupled to Ni^{2+}-coated biosensors and loaded with 0.5 μM of non-tagged Sos1 (association phase). Subsequently, dissociation of Sos1 was monitored in buffer in the presence or absence of 5 mM Fru6P (**a**), DHAP (**b**), Glucose (**c**), GAP (**d**) or GTP (**e**). The rates of Sos1 dissociation were strongly increased in the presence of Fru1,6bisP at physiological concentrations, while Fru6P and DHAP only produced a very small effect at non-physiological concentrations (**f**).

Fig 4a - Ras-GTP

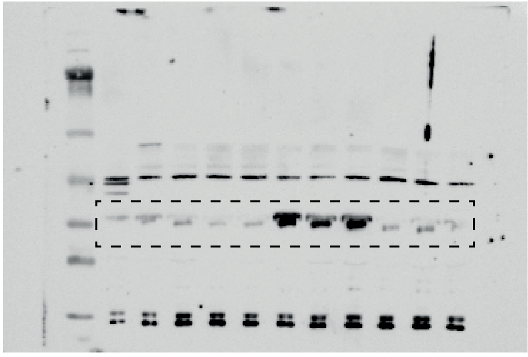

Fig 4a - Total Ras

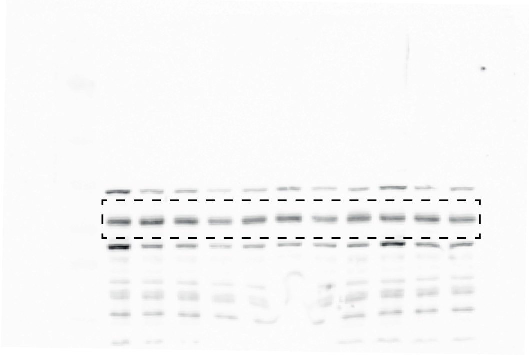

Fig 5b - Ras-GTP (top) & Total Ras (bottom)

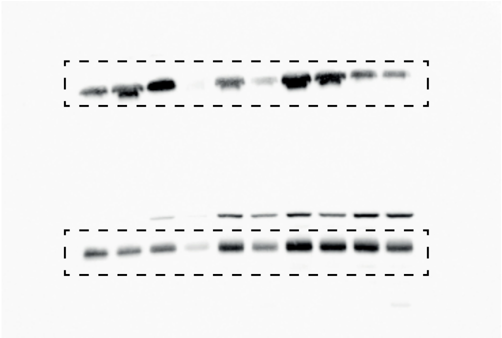

Fig 5c - Ras-GTP (top) & Total Ras (bottom)

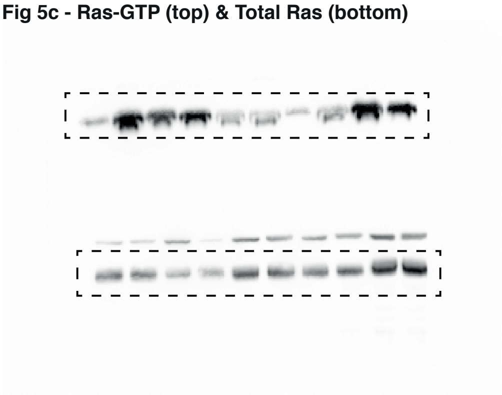

Supplementary Fig. 12

Uncropped images of key western blot figures shown in the main manuscript. Note that the western blots shown in the uncropped images for Fig 5b and 5c are obtained by combining the relevant parts of two gels together on one nitrocellulose membrane prior to western blotting.

Supplementary Table 1: list of strains

Yeast strains	Relevant genotype	Reference
W303-1A (wild type)	*Mata leu2-3,112 ura3-1 trp1-1 his3-11,15 ade2-1 can1-100 GAL SUC*	[1]
YSH290	*tps1::TRP1*	[2]
YSH312	*tps1::TRP1 hxk2::LEU2*	[2]
JT22287	*tps1::TRP1 ras2::KanMX*	This work
KEN9	*ira1:: KanMX ira2::KanMX*	This work
KEN22	*tps1::TRP1 pfk1::KanMX*	This work
KEN24	*tps1::TRP1 pfk2::KanMX*	This work
KEN25	*tps1::TRP1 pfk1:: KanMX pfk2::KanMX*	This work
KEN27	*tps1::TRP1 ira1:: KanMX ira2::KanMX*	This work
KEN29	*ira1:: KanMX ira2::KanMX cdc25::LEU2*	This work
KEN31	*tps1::TRP1 ira1:: KanMX ira2::KanMX cdc25::LEU2*	This work
KEN33	*ira1:: KanMX ira2::KanMX sdc25::HIS3*	This work
KEN35	*tps1::TRP1 ira1:: KanMX ira2::KanMX sdc25::HIS3*	This work
KEN37	*ira1:: KanMX ira2::KanMX cdc25::LEU2 sdc25::HIS3*	This work
KEN39	*tps1::TRP1 ira1:: KanMX ira2::KanMX cdc25::LEU2 sdc25::HIS3*	This work
KEN46	*cdc25::CDC25*^{T1490P}	This work
KEN50	*cdc25::CDC25*^{K1491E}	This work

KEN52	*cdc25::CDC25*^{R1122D}	This work
KEN53	*cdc25::CDC25*$^{K1491E\ R1122D}$	This work
KEN58	*cdc25::SOS1*$_{553\text{-}1024}$	This work
KEN59	*cdc25::SOS1*$_{553\text{-}1024}^{R962T}$	This work
KEN61	*cdc25::SOS1*$_{553\text{-}1024}^{R962P}$	This work
KEN64	*cdc25::SOS1*$_{553\text{-}1024}^{K963E}$	This work
KEN67	*cdc25::SOS1*$_{553\text{-}1024}^{K602E}$	This work
KEN68	*cdc25::SOS1*$_{553\text{-}1024}^{K602E\ K963E}$	This work
